# HelixDiff, a Score-Based Diffusion Model for Generating All-Atom α-Helical Structures

**DOI:** 10.1021/acscentsci.3c01488

**Published:** 2024-04-05

**Authors:** Xuezhi Xie, Pedro A Valiente, Jisun Kim, Philip M Kim

**Affiliations:** † Donnelly Centre for Cellular and Biomolecular Research, University of Toronto, Toronto, Ontario M5S 3E1, Canada; ‡ Department of Computer Science, University of Toronto, Toronto, Ontario M5S 3E1, Canada; § Department of Molecular Genetics, University of Toronto, Toronto, Ontario M5S 3E1, Canada

## Abstract

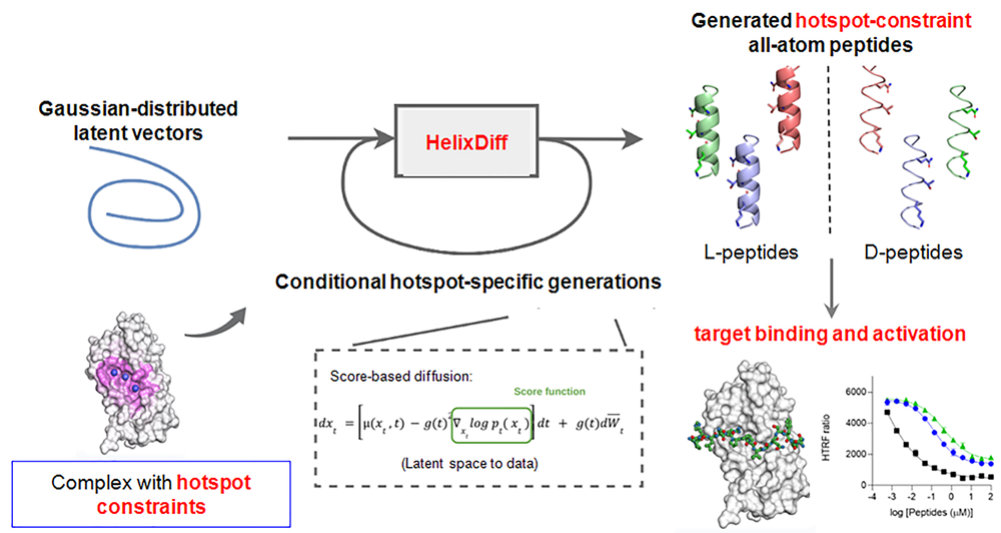

Here, we present HelixDiff, a score-based diffusion model for generating all-atom helical structures. We developed a hot spot-specific generation algorithm for the conditional design of α-helices targeting critical hotspot residues in bioactive peptides. HelixDiff generates α-helices with near-native geometries for most test scenarios with root-mean-square deviations (RMSDs) less than 1 Å. Significantly, HelixDiff outperformed our prior GAN-based model with regard to sequence recovery and Rosetta scores for unconditional and conditional generations. As a proof of principle, we employed HelixDiff to design an acetylated GLP-1 D-peptide agonist that activated the glucagon-like peptide-1 receptor (GLP-1R) cAMP accumulation without stimulating the glucagon-like peptide-2 receptor (GLP-2R). We predicted that this D-peptide agonist has a similar orientation to GLP-1 and is substantially more stable in MD simulations than our earlier D-GLP-1 retro-inverse design. This D-peptide analogue is highly resistant to protease degradation and induces similar levels of AKT phosphorylation in HEK293 cells expressing GLP-1R compared to the native GLP-1. We then discovered that matching crucial hotspots for the GLP-1 function is more important than the sequence orientation of the generated D-peptides when constructing D-GLP-1 agonists.

## Abstract 2

We developed a score-based diffusion model for generating all-atom α-helix structures and enabling conditional peptide design by mimicking critical hotspot residues in bioactive peptides.

## Introduction

Computational protein design holds tremendous potential within biomedicine, spanning diagnostics, biosensors, therapeutics, and synthetic biology.^1^ At the core of its significance are protein–peptide interactions, pervasive within molecular pathways, deeply influencing cellular functions by orchestrating up to 40% of protein–protein interactions.^2^ Peptides, marked by attributes conducive to therapeutic progress, boast heightened specificity and reduced toxicity relative to small molecules.

α-Helix peptides, constituting the most prevalent secondary structure in proteins, play a pivotal role in conferring stability, comprising about 30% of the average globular protein’s architecture.^3^ Notably, α-helical peptides are involved in nearly 40% of homodimeric and 26% of heterodimeric protein–protein interfaces.^4^ Despite their prominence, native α-helical peptides pose challenges as drug candidates due to their diminished conformational stability in the absence of a protein scaffold and their susceptibility to proteolysis.^5^ In contrast, peptides composed of D-amino acids exhibit distinctive advantages, including low immunogenicity, cost-effective manufacturing, and robust proteolytic stability.^6−8^

We previously developed an in-house methodology that converts (L)-peptides into highly stable D-analogues through a mirror-image search in the protein data bank (D-PDB)^9^ and a deep learning approach called HelixGAN for *de novo* design.^10^ Using both methods, we have designed retro-inverted D-peptide analogues capable of activating the GLP-1, PTH,^9^ and GLP-2 receptors^11^ while also inhibiting SARS-CoV-2 infections *in vitro*.^12,13^ However, the D-PDB database encompasses a relatively substantial collection of native helical structures, representing a fraction of the possible stable helices, while the sample generation and indirect search for hotspot design limit the HelixGAN model.

Diffusion models have recently gained popularity in the field of biology. These models have outperformed GAN, the top generative model of the past decade, at finding intricate patterns within large data sets and creating synthetic data with desired attributes. Protein design diffusion approaches currently focus on building protein backbone structures without considering sequence information.^1,14^ These algorithms require additional techniques, such as inverse folding, to predict the sequence for the generated structure.^15,16^ Due to the inclusion of other methodologies, this process could result in suboptimal designs and errors.

This article presents HelixDiff, a score-based diffusion model that directly generates sequence and structural features in end-to-end training processes, mirroring patterns found in real helices. We developed a hotspot-specific generation algorithm for the conditional design of α-helices targeting critical hotspot residues in bioactive peptides. As a proof of concept, we created an acetylated GLP-1 D-peptide agonist (D-GLP-1_diff_Acetylated) that selectively activated the GLP-1R by matching critical hotspots in GLP-1. D-GLP-1_diff_Acetylated stimulated HEK293 cells transfected with the GLP-1 receptor with an EC_50_ of 413 nM, which makes it less potent than GLP-1 (EC_50_ = 0.18 nM). This D-peptide agonist is highly resistant to protease degradation and selectively activates the GLP-1 receptor without stimulating GLP-2 receptor signaling. Notably, despite its lower potency, this analogue enhanced the AKT phosphorylation at the same levels as the native GLP-1. We discovered that matching crucial hotspots for the GLP-1 function is more critical than the sequence orientation of the generated D-peptides when developing D-GLP-1 agonists. We envision HelixDiff as an essential tool for creating novel bioactive peptides with specified features in the early stages of drug discovery.

## Results

### HelixDiff, a Score-Based Diffusion Model for the Full-Atom Design of α-Helical Peptide Structures

**Figure 1:**
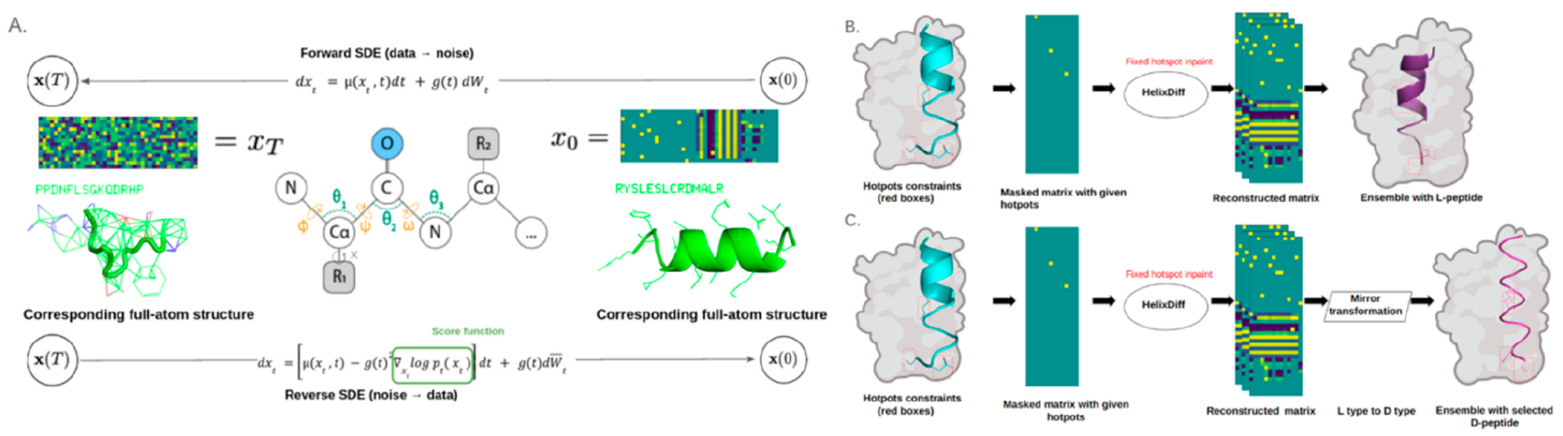
Flowchart of the de novo methodology for generating helical structures coined as HelixDiff. (A) Flowchart of the score-based diffusion model. The model is trained to create realistic helical structures from noise by learning a reverse “denoising” process given the forward diffusion process that maps data to Gaussian noise. The feature map in the middle shows the encoded information to generate the full-atom structures, including the sequence and structural information (angle) vectors, respectively. The sequence is represented using one-hot encoding, and the angle vector includes ϕ, ψ, ω, bond angles, and χ angles. (B, C) Hotspot-specific generation module flowchart. The module is given only the hotspot positions and amino acid types as shown. Then, the hotspot-specific generation module generated all the rest of the regions, as shown in the reconstructed matrix. The model can also generate D-helical peptides by transforming the generated L-helices into D-peptides using a mirror transformation (as shown in (C)).

We developed HelixDiff, a score-based diffusion model designed for generating full-atom helices. These helices are characterized by image-like representations, combining one-hot encoded sequence data with structural information, encompassing torsional angles, bond angles, planar angles, and side-chain information, as depicted in Figure S1. We previously used this encoding scheme to generate all-atom model α-helix structures using HelixGAN.^10^ Utilizing the U-Net architecture for the scoring network,^17^ we established a framework for designing full-atom peptides, as illustrated in Figure 1A.

We next integrated a conditional hotspot-specific generation module that is tailored explicitly to the receptor of interest as shown in Figure 1B,C. We defined hotspot residues as those that have a critical contribution for target recognition, binding, and receptor activation. We then constrained the structural generation of the novel peptides to a set of identified hotspots to produce functional designs. The hotspot residue information served as contextual cues for the module to reconstruct the remaining data. The conditional generation process focused on fulfilling the encoding matrix, resulting in more targeted and precise generations. We could generate a variety of realistic conformational rotamers based on hotspot residues and amino acid types, resulting in superior conformation matching, especially in D-peptide design settings.

**Figure 2:**
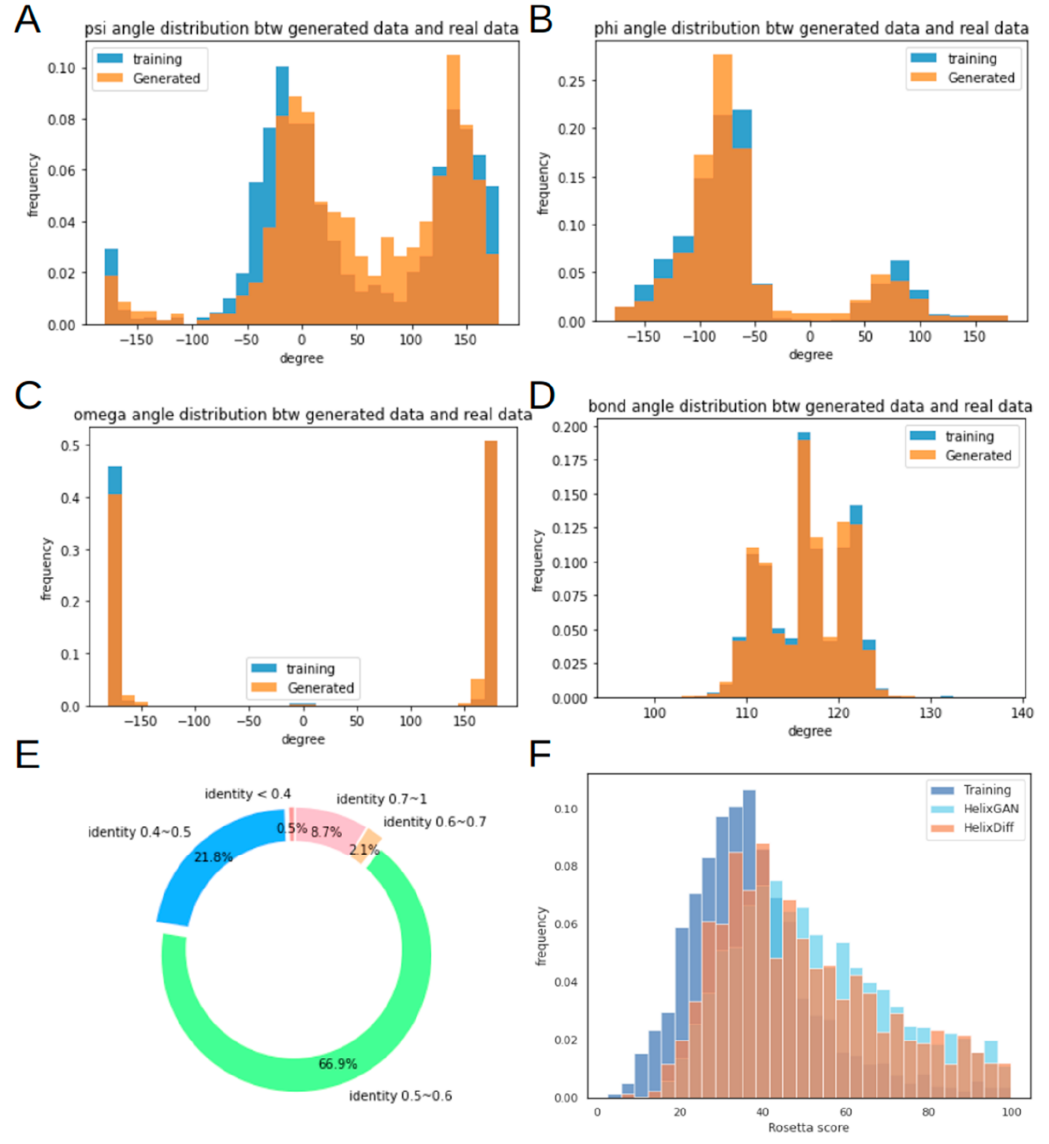
Assessment of novel helices generated with HelixDiff. (A–D) Structural features regarding ϕ, ψ, ω,, and bond angles between 2k generated samples and training data. (E) Sequence identities were compared with training data. (F) Comparing the Rosetta score distribution of the training data with those generated with HelixGAN and HelixDiff.

Following that, we unconditionally generated synthetic helical structures using HelixDiff of 14 amino acids and compared our results with HelixGAN. We evaluated the structure’s quality by randomly unconditionally sampling 2000 helices using HelixDiff and HelixGAN. The generated data contain a range of physical structural features similar to that of the training data (Figure 2A–D). Notably, most of the generated data (77.7%) exhibited a sequence identity with the training data above 50% outperforming HelixGAN (Figure 2E and Figure S2). We then compared the structural similarity of HelixDiff’s α right-handed helix structures to PEP-FOLD4,^18^ a commonly used algorithm for predicting peptide 3D structures. For a small random sample of sequences, we found a strong resemblance between the structures generated by both techniques (Figure S3).

**Figure 3:**
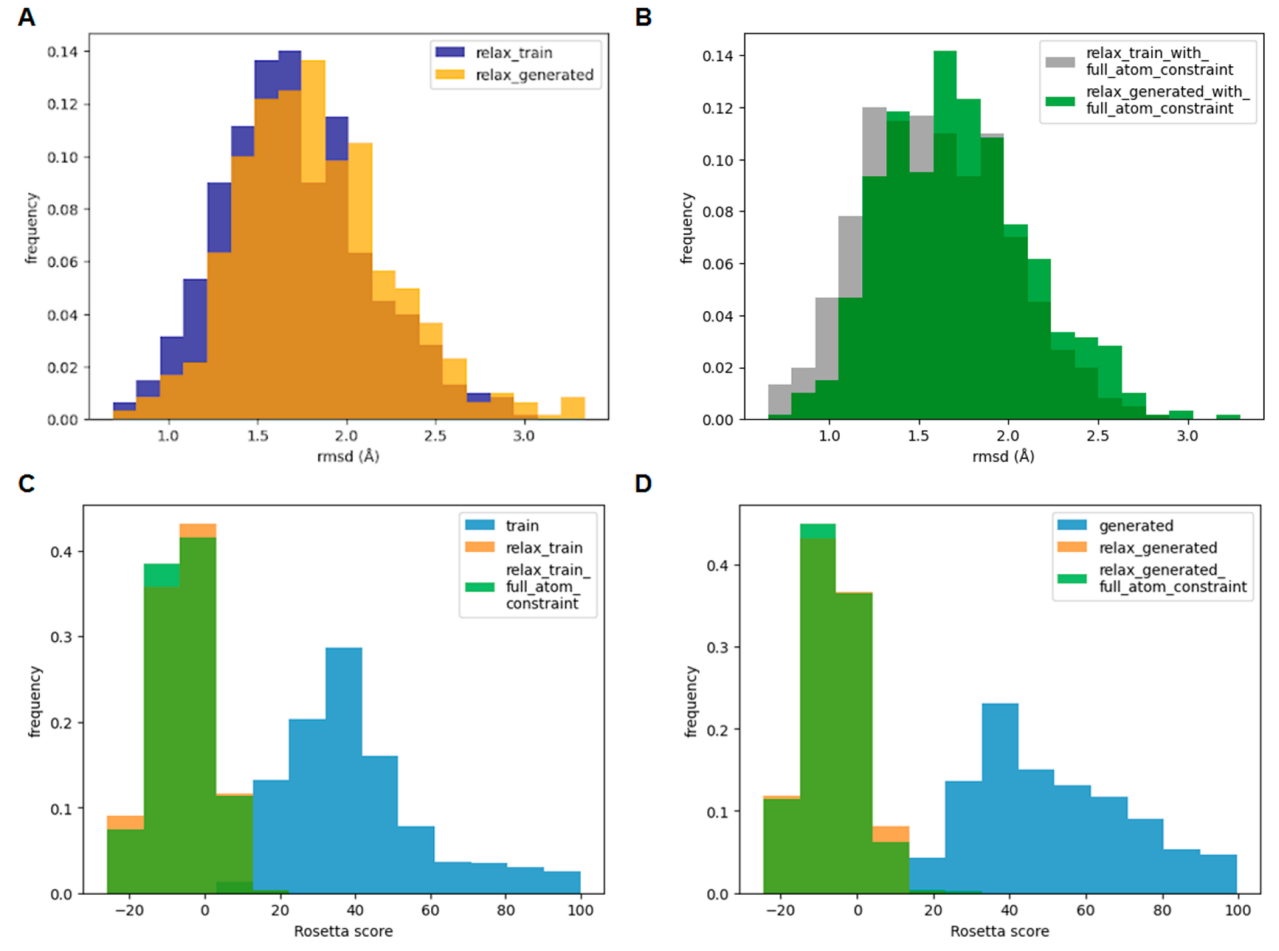
rmsd and Rosetta score distributions of the generated structures before and after Rosetta relaxation. (A) rmsd distribution of the structures in the training and generated data sets after relaxation without constraints. (B) rmsd distribution of the structures in the training and generated data sets after relaxation by using all-atom constraints. (C) Rosetta score distributions of the structures in the training set without relaxation (blue), after relaxation without constraints (orange), and after relaxation with constraints (green). (D) Rosetta score distributions of generated structures without relaxation (blue), after relaxation without constraints (orange), and after relaxation with constraints (green). We randomly collected 600 structures from the training and generated data. Following the Rosetta relaxation protocol, we calculated the rmsd between the structures before and after relaxation. The rmsd values were calculated considering all the atoms in each structure.

We also compared the Rosetta score distribution obtained with HelixDiff and HelixGAN for the training and generated data. HelixDiff showed a more similar distribution to training data than HelixGAN, indicating a better performance for α-helix structure generation (Figure 2F). However, the Rosetta scores observed for all structures were positive, implying that the resulting designs include internal clashes or distortions in bond geometry. We then randomly picked 600 structures from the generated and training data sets for minimization with the Rosetta force field^39^ as an additional validation (Figure 3). To conduct a thorough comparison, we measured both main chain and all-atom RMSD (Table 1), as the precise generation of side-chain conformations is critical in our approach. Our analysis showed similar RMSD distributions for training and generated data after Rosetta energy minimization with and without constraints (Figure 3A,B), with median and average values ranging from 0.1 to 0.2 Å. Significantly, after energy minimization with the Rosetta force field, the scores obtained for most structures were negative (Figure 3C,D). This result suggests that our generated data exhibit chemical properties comparable to those of experimental data, bolstering the validity of our results.

**Table 1:** rmsd of the Generated and Training Structures after Minimization with Rosetta Relax^a^

	rmsd relax for generated data (Å)		rmsd relax for training data (Å)	
	no constraints	constraints	no constraints	constraints
main chain (avg)^b^	0.89	0.82	0.77	0.70
main chain (med)^c^	0.85	0.78	0.73	0.65
all atom (avg)^b^	1.82	1.72	1.70	1.59
all atom (med)^c^	1.78	1.68	1.65	1.56

a We calculated the rmsd after the relaxation using the Rosetta relax protocol for each structure, considering the main chain and all atoms.

b Average rmsd of a set of structures.

c Median rmsd of a set of structures.

We next assessed the performance of our hotspot-specific generation module on the test set, generating L-type helices that match desired hotspot residues (Table 2). The evaluation involved calculating the rmsd between the target and generated helix structures via partial alignment of the hotspots and matched residue atoms. Impressively, in 54.5% of the 3-hotspot test cases, the RMSDs of matched residues in the newly generated helices were less than 1 Å for the target hotspots. At the same time, 31.1% fell between 1 and 1.5 Å (Table 2 and Figure S4A–C). The Rosetta scoring function classified most of the generated helix structures as reasonable (Figure S4D), which is better than that of HelixGAN.

**Table 2:** Evaluation of the Hotspot-Specific Conditional Generation with HelixDiff and HelixGAN^a^

type	hotspots	method	rmsd (<1, %)	rmsd (1–1.5, %)	rmsd (1.5–2, %)	rmsd (2–5, %)	NA (%)
L type	3	HelixGAN	7.8	23.3	34.4	22.2	12.2
D type	3	HelixDiff	54.5	31.1	11.1	3.3	0
							
L type	3	HelixGAN	0	32.6	40.4	3.4	23.6
D type	3	HelixDiff	10	48.9	37.8	3.3	0
							
D type	4	HelixDiff	1.4	34.7	55.6	8.3	0

a For every two rows, the same test cases were performed between HelixGAN and HelixDiff. The lowest rmsd for each test case is calculated and summarized in the table. The hotspot columns indicate the randomly selected hotspot residues in the test cases. Noticed that HelixGAN could not perform 4-hotspot conditional generations.

One of our primary objectives is to use our model for designing D-peptides, mimicking bioactive L-peptides. We applied a mirror conversion step to transform the helices generated with HelixGAN into D-peptides. Given a set of hotspots in a known L-peptide, our model conditionally generates novel D-helix peptide structures using an in-painting mechanism and mirror conversion. In 58.9% of the 3-hotspot test cases, the RMSDs of matching residues were below 1.5 Å, while 10% fell below 1.0 Å (Table 2 and Figure S5A–C). Most novel D-helix structures received favorable classifications based on their Rosetta scores (Figure S5D). We also tested our model to generate D-peptides using 4-hotspot test sets. HelixDiff could generate D-peptides with matching residues below 1.5 A in 36.1% of the 4-hotspot test cases (Figure S5E). This new model significantly outperformed HelixGAN, which could not support the 4-hotspot D-peptide design due to the inefficiencies of the indirect search (Figure S5F).

### *De Novo* Design of a D-peptide Agonist of the GLP-1 Receptor Using HelixDiff

**Figure 4:**
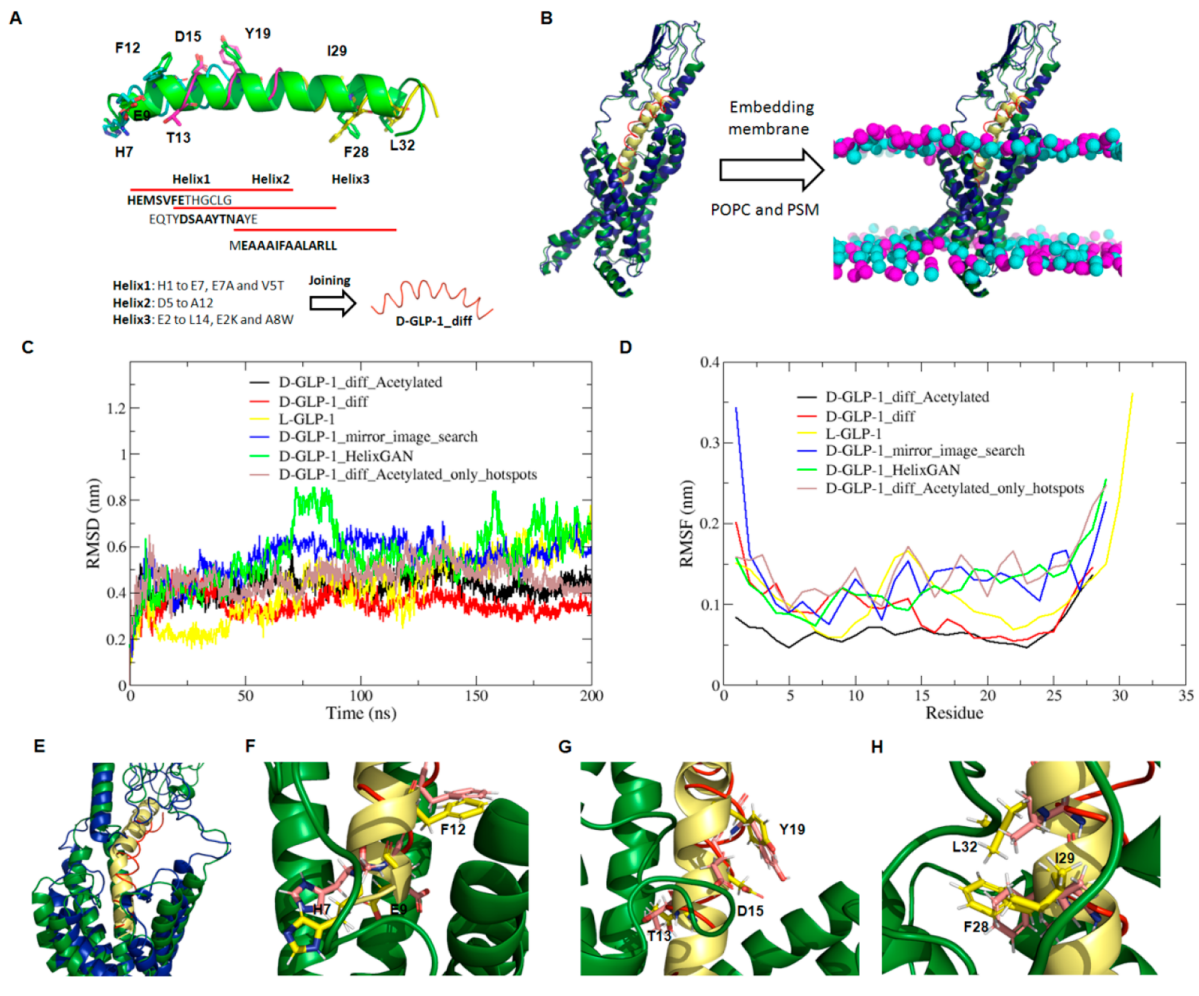
Design and modeling of the 3D structure of D-GLP-1_diff analogues bound to the GLP-1 receptor. (A) Strategy for designing two novel D-GLP-1 analogues using HelixDiff. The GLP-1 structure was divided into three overlapping peptides named helixes1–3. Helix1 extends from H7 to L20 and helix2 runs from F12 to A25, while helix3 extends from Q23 to R36. Critical hotspots for the GLP-1 function chosen for the design were colored as licorice. (B) Structural superposition of D-GLP-1_diff analogue (red) over the L-GLP-1 (yellow) structure bound to the GLP-1 receptor (pdb code: 5vai). In dark green is shown the GLP-1R coupled to GLP-1, while in dark blue is represented the receptor bound to the D-peptide analogue. The magenta and cyan spheres represent the phosphate groups of the lipids polar heads. (C) Root mean square deviation (rmsd) of the heavy atoms of L-GLP-1 and the D-GLP-1 analogues bound to the GLP-1R. (D) Root mean square fluctuation (RMSF) per residue of the heavy atoms of L-GLP-1 and the D-GLP-1 analogues bound to the GLP-1R. (E) Structural superposition of the most representative cluster extracted from the MD simulation of GLP-1R bound to D-GLP-1_diff_Acetylated over the experimental structure of the GLP-1R in complex with GLP-1 (5vai). The D-GLP-1_diff_Acetylated analogue is shown in red, while GLP-1 is displayed in yellow. In dark green is shown the GLP-1R coupled to GLP-1, while in dark blue is represented the receptor bound to the D-peptide analogue. (F) Enlargement of the structural alignment of D-GLP-1_diff_Acetylated (red) with GLP-1 (yellow) at helix1. We showed as licorice the hotspots and matching residues (red) in the L-peptide (yellow) and D-peptide (red), respectively. (G) Enlargement of the structural alignment of D-GLP-1_diff_Acetylated (red) with GLP-1 (yellow) at helix2. We showed as licorice the hotspots and matching residues (red) in the L (yellow)- and D-peptide (red), respectively. (H) Enlargement of the structural alignment of D-GLP-1_diff_Acetylated (red) with GLP-1 (yellow) at helix3. We showed as licorice the hotspots and matching residues (red) in the L (yellow)- and D-peptide (red), respectively. In the hotspots and matching residues, the nitrogen and oxygen atoms are colored in blue and red, respectively.

Here, we targeted a set of well-known hotspot residues of GLP-1 to design a novel GLP-1 D-peptide analogue using HelixDiff (Table S1). To generate a D-GLP-1 analogue, we used the ligand structure in complex with the full-length GLP-1 receptor as a starting point (PDB code: 5vai).^19^ Before designing the D-peptide analogue, we divided the GLP-1 structure into three overlapping fragments named helix1, helix2, and helix3. Helix1 extends from H7 to L20 and helix2 runs from F12 to A25, while helix3 runs from Q23 to R36. Positions H7, E9, and F12 were designated as hotspots in helix1. These residues are located in the GLP-1 region, interacting with the GLP-1 receptor’s transmembrane (TM) core.^19^ H7 and F12 are conserved, whereas E9 retains the negative charge of the corresponding site in GLP-2 or glucagon.^20^ For helix2, T13, D15, and Y19 were selected as hotspots. T13 is conserved in glucagon, keeps the polarity of the equivalent position in GLP-1, and primarily interacts with TM2 and TM7.^19^ D15 is conserved in glucagon, similar to E9 in GLP-2, and binds to TM1 and ECL1, while Y19 is conserved in glucagon, keeping the hydrophobic nature of I13 in GLP-2 and targeting the ECL1.^20^ In the case of helix3, we chose residues F28, I29, and L32 (Figure 4A). These residues bind to the extracellular domain of the GLP-1 receptor.^19^ F28 and L32 are conserved in GLP-1 and glucagon, respectively, while I29 is conserved in GLP-2 while retaining the nonpolar nature of the GLP-2 equivalent site.^20^ Several D-helix structures were generated after running HelixDiff for each helix independently. The root-mean-square deviation (rmsd) between the specific atoms in each starting helix structure and the generated D-helix structures was used to evaluate the match quality. We next joined the best-matched peptides generated to design D-GLP-1_diff (Figure 4A).

We then superimposed the D-GLP-1_diff structure onto the Cryo_EM structure of GLP-1R bound to GLP-1 to build the 3D structure of the GLP-1 receptor (GLP-1R) in complex with the novel D-peptide analogue (Figure 4B). The GLP-1R+D-GLP-1_diff complex was then embedded in a POPC: PSM (1:1) bilayer before evaluating its binding mode stability using 200 ns MD simulations (Figure 4B). We also simulated the GLPR1 bound to D-GLP-1_diff acetylated in the N-terminal, the wild-type L-GLP-1, and the D-GLP-1 peptides designed earlier after scanning a mirror-image version of the protein data bank (D-PDB)^9^ and using HelixGAN.^10^ The peptides designed in the current work (D-GLP-1_diff and D-GLP-1_diff_Acetylated) share the same orientation with the L-peptide and D-GLP-1_HelixGAN. In contrast, the D-GLP-1_mirror-image peptide is retro-inverted compared to the L-peptide.^9^ This difference in the direction means that D-GLP-1_diff peptides have their N-terminal residue embedded in the transmembrane domain of the GLP-1 receptor, while the D-GLP-1_mirror-image peptide has its N-terminal residue interacting with the extracellular domain of the receptor. Like the L-GLP-1, all D-peptides quickly stabilized in a new equilibrium position close to the initial structure, according to the rmsd profiles calculated for the peptide’s heavy atoms (Figure 4C). The calculated RMSF profiles revealed that both D-GLP-1_diff analogues were more stable than the one created using HelixGAN (Figure 4D).

To predict the impact over the peptide binding mode stability of mutating all the nonhotspot residues to alanine, we designed a polyalanine D-peptide carrying the same hotspots named D-GLP-1_diff_Acetylated_only_hotspots. As expected, the D-GLP-1 analogue with all the nonhotspot residues mutated to alanine showed a more significant structural fluctuation than the wild-type D-peptide (Figure 4D). Nonhotspots differed significantly between the original GLP-1 and the D-GLP-1 analogues (Table S1). Although these differences may not play a significant role in this interaction, they may harm binding. These mutations will make the peptide more hydrophobic and could hinder the peptide’s solubility and functional activity. The structural superposition of the most representative clusters extracted from the MD simulation of the GLPR1+D-GLP-1_diff_Acetylated and GLPR1+L-GLP-1 experimental structure revealed a significant matching between specific residues in the D-GLP-1_diff_Acetylated analogue and the majority of the hotspots defined in L-GLP-1 (Figure 4E–H). The most significant differences were observed for H7 and E9, two important hotspots in the N-terminal segment region of L-GLP-1 (Figure 4F).^21^

### Experimental Validation of the D-GLP-1 Peptide Designed with HelixDiff

**Figure 5:**
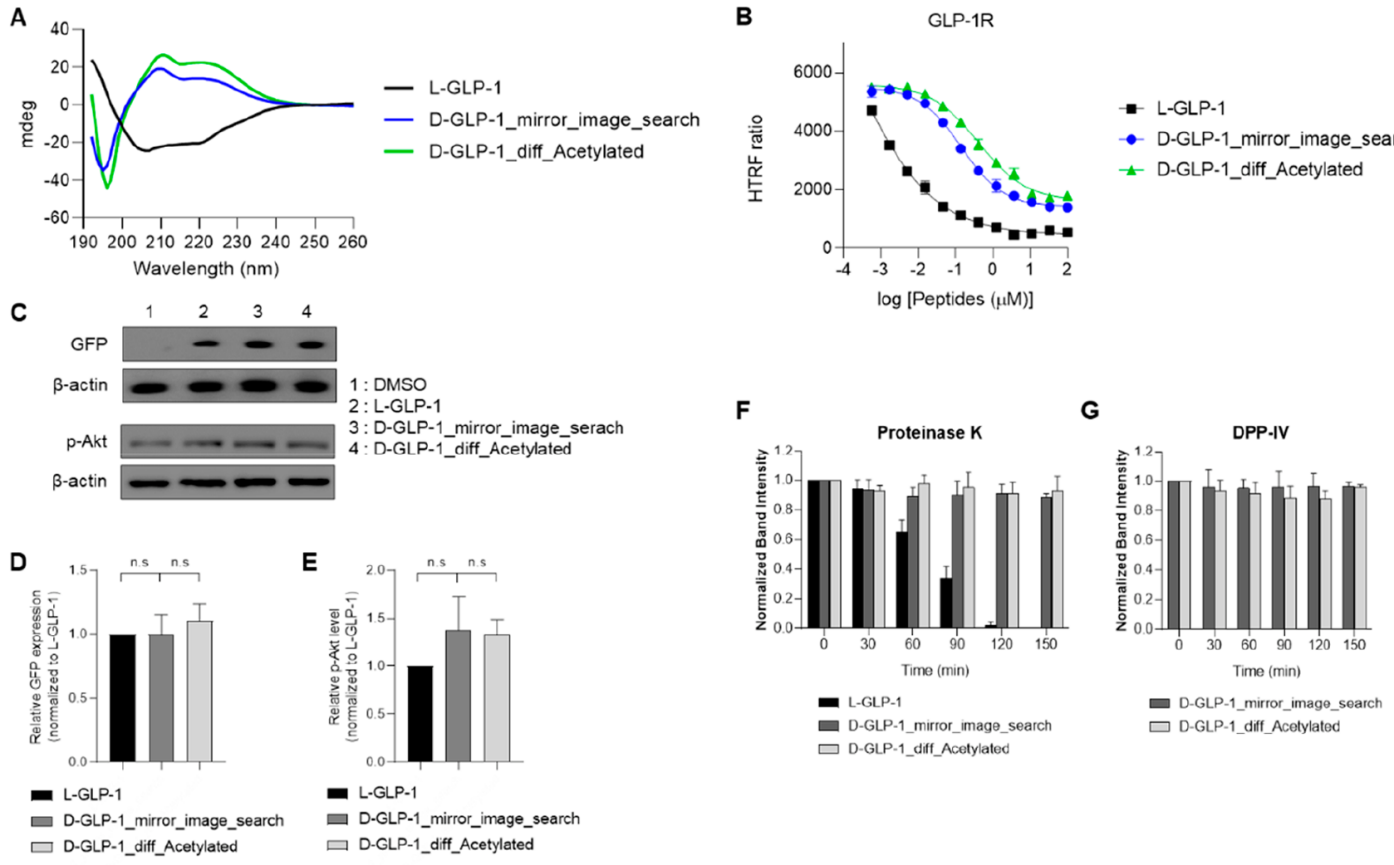
Experimental validation of the D-GLP-1 agonist designed with HelixDiff. (A) Circular dichroism measurements of the D-GLP-1 designs and L-GLP-1 in solution. All peptides were dissolved in acetonitrile:PBS (1:2). (B) Activity profile of L-GLP-1 and the D-GLP-1 peptides over HEK293 cells stably expressing GLP-1R and CRE-luciferase. (C) Western blots showing the GFP and p-AKT expression levels induced by the D-GLP-1 analogues and the native L-GLP-1 at 10 μM. The DMSO sample is a negative control, and β-actin expression in all the treatments is a loading control. (D) Quantification of the GFP expression levels induced for the D-GLP-1 agonists relative to L-GLP-1 treated cells after 36 h of incubation. n.s.: not significant. (E) Quantification of the AKT phosphorylation levels induced for the D-GLP-1 agonists relative to L-GLP-1 treated cells after 4 h of incubation. n.s.: not significant. (F) Quantification of the remaining D-GLP-1 analogues and L-GLP-1 after Proteinase K treatment in 30 min intervals. Intensities of the peptide bands were normalized to the intensity of the untreated peptide (T0). (G) Quantification of the remaining D-GLP-1 analogues after DPP-IV treatment in 30 min intervals. Intensities of peptide bands were normalized to the intensity of the untreated peptide (T0). The SDS-PAGE gel images are shown in Figure S8.

To experimentally validate the designed D-GLP-1 peptide, D-GLP-1_diff_Acetylated was chemically synthesized. As a control, L-GLP-1 peptide and D-GLP-1_mirror_image_search,^9^ a D-GLP-1 analogue designed earlier after searching a mirror-image version of the protein data bank (D-PDB), were synthesized as well. We then performed circular dichroism analysis of the peptides in solution to determine the peptides’ secondary structure. According to our findings, all D-peptides and L-GLP-1 remain helical in solution (Figure 5A).

Following that, we generated a stable GLP-1R-expressing HEK293 cell line to test the ability of the L-GLP-1 and D-GLP-1_diff_Acetylated designs to activate the GLP-1R. GLP-1 binding to GLP-1R has previously been reported to activate adenylyl cyclase to produce cyclic adenosine monophosphate (cAMP), which stimulates protein kinase A (PKA) that plays an essential role in a variety of signaling pathways. To assess the potency of L-GLP-1 and the D-peptides, we used the HTRF cAMP assay. This methodology identifies intracellular cAMP by competing for the anti-cAMP antibody with d2-labeled cAMP following cell lysis. The FRET signal is then disrupted as intracellular cAMP accumulates. L-GLP-1 decreased the FRET signal in GLP-1R expressing HEK293 cells with a half maximal effective concentration (EC_50_) value of 0.18 nM. D-GLP-1_mirror_image_search and D-GLP-1_diff_Acetylated designs reduced the FRET signal to a lesser extent, with EC_50_ values of 142 and 413 nM, respectively. Both D-GLP-1 agonists stimulated the GLP-1R with efficacies ranging from 71.6 to 73.9% compared to L-GLP-1 activation (Figure 5B).

We next tested the specificity of the D-GLP-1 analogues to activate the GLP-2R using a GLP-2R-expressing HEK293 cell line. Notably, neither the D-GLP-1 analogues nor the L-GLP-1 peptide could activate the GLP-2R signaling, demonstrating their specific GLP-1R recognition (Figure S7). GLP-2R was chosen for the specificity assays over the other members of the B-class GPCR receptor subfamily due to its higher percentage of sequence identity with the GLP-1R.^22^ We considered that because these analogues could not stimulate the GLP-2R, they would not activate other members of this subfamily.^22^

We then investigated the downstream effects of activating the GLP-1R with the D-GLP-1 analogues. We determined whether stimulating the GLP-1R with the D-GLP-1 analogues would promote GFP expression in HEK293 cells containing a GFP gene under the control of cAMP response element binding protein (CREB) and AKT phosphorylation. We also checked the β-actin expression in all of the treatments as loading controls. Of relevance, all peptides induced a similar GFP expression under the CREB following 36 h of incubation (Figure 5C,D). Furthermore, both D-GLP-1 analogues triggered slightly higher phosphorylation levels of AKT to L-GLP-1 in HEK293 cells expressing GLP-1R, but not significantly (Figure 5C,E).

Finally, we examined the resistance of both D-GLP-1 analogues to the proteinase K degradation and DPP-IV cleavage (Figure 5F,G and Figure S8). Endogenous GLP-1 is rapidly cleaved by the DPP-IV enzyme, thereby losing its biological activity. In terms of therapeutic applications, D-peptides have the advantage of protease resistance since it translates into a longer half-life in serum. L-GLP-1 was almost completely degraded in 2 h, while over 88% of both D-GLP-1 analogues can remain after 2.5 h of proteinase K exposure (Figure 5F and Figure S8A). Similarly, both D-GLP-1 analogues were stable after 2.5 h of incubation with DPP-IV (Figure 5G and Figure S8B). Consequently, the D-GLP-1 agonists showed high protease resistance, thereby expecting a longer half-life and higher therapeutic potency.

## Discussion

Here, we present HelixDiff, our pioneering score-based diffusion network method to design novel full-atom helical structures from the ground up. While previous diffusion methods primarily focused on generating protein backbone structures without considering sequence information,^14,1,16^ our methodology directly generates sequence and structural features in end-to-end training processes, mirroring patterns found in real helices. We implemented a hotspot-specific generation algorithm^23^ for the conditional design of α-helices targeting critical hotspot residues in bioactive peptides. When we compared HelixDiff to HelixGAN, our prior GAN model,^10^ we noticed different strengths and performance differences. Although both models are designed for all-atom peptide generation and GANs are often thought to be more computationally efficient, HelixDiff, which functions as a score-based generative diffusion model, outperforms HelixGAN in several ways. HelixDiff consistently demonstrates superior performance in unconditional generation tasks, producing peptide structures with enhanced structural quality. Significantly, the diffusion model provides nuanced control over the generation process, with HelixDiff excelling in crafting peptides tailored to specific requirements such as aligning with critical hotspots in known structures.

Hotspot residues are critical in molecular recognition, receptor activation, and therapeutic development.^24^ Here, we developed a hotspot-specific generation algorithm for the conditional design of α-helices targeting critical hotspot residues with HelixDiff. We employed a different test data set with randomly selected hotspot residues in L-peptides to check if our model could build helical structures matching hotspot residue conformations. Notably, HelixDiff can generate unique helices that correspond to L-type hotspot residues. However, native α-helical peptides are poor therapeutic candidates because of their low structural stability in the absence of the protein scaffold and high vulnerability to proteolysis.^25^

HelixDiff outperformed our previously developed generative adversarial network method to create D-peptides matching a set of specific hotspots in a known bioactive L-peptide. This finding is essential for extending the structural space for designing D-peptides from scratch, as it overcomes the constrained conformational space of helix structures deposited at the PDB and generated by GAN models such as HelixGAN. We then used HelixDiff to develop a novel D-peptide analogue that bound the GLP-1R and matched the conformations of critical hotspots for the GLP-1 function. GLP-1 was chosen as a study example, since it is now being studied as diabetes and obesity therapy.^26,27^ Earlier theoretical and experimental studies supported our GLP-1 hotspot residue selection.^9,28^ Our MD simulations showed a stable binding mode of the D-GLP-1_diff_Acetylated coupled to the GLP-1R. This novel D-peptide analogue has a orientation similar to that of GLP-1 and is substantially more stable in MD simulations than our earlier D-GLP-1 designs. The differences in the RMSF profile between the D-GLP-1_diff analogues and the D-GLP-1 agonist developed using a mirror-image search in the protein data bank (D-PDB) are due to differences in peptide orientation.

The D-GLP-1_diff_Acetylated agonist activated the GLP-1R signaling with an EC_50_ value weaker than that of the native L-GLP-1 but similar to that of our previous D-GLP-1_mirror_image_search design. We used almost the same collection of hotspot residues in the GLP-1 sequence to design both D-peptides (Table S1). We also demonstrated that matching critical hotspots for the GLP-1 function is more relevant to retaining the GLP-1’s agonist effect than the sequence orientation of the D-peptide analogue. Similarly to our previous study,^9^ we confirmed that the GLP-1 sequence could be changed entirely except for hotspots. We previously showed that a scrambled form of the D-GLP-1_mirror_image_search peptide was inactive.^9^ Thus, we hypothesized that a scrambled variant of D-GLP-1_diff_Acetylated would be inactive given that we designed both D-peptides using a similar set of hotspots in the GLP-1 sequence. Further experiments are needed to confirm this prediction.

We then used short-term and long-term stimulation experiments to evaluate the downstream impact of stimulating the GLP-1R with the D-GLP-1_diff_Acetylated agonist with our earlier D-GLP-1 design and the native GLP-1. As a short-term study, we assessed the increase of the p-AKT levels after the GLP-1R stimulation for 4 h. The novel D-GLP-1 analogue triggered the p-AKT levels like L-GLP-1 and the former D-GLP-1 variant did. The GLP-1R’s more prolonged activation could explain the enhancement in the p-AKT levels induced for the D-GLP-1 analogues. As a long-term stimulation study, we detected the GFP expression under the control of cAMP response element binding protein for 36 h. The more prolonged activation of the GLP-1R could compensate for the lower levels of cAMP induced by both D-GLP-1 analogues, making them induce GFP expression levels similar to those for L-GLP-1.^29^ More experiments are needed in the future to confirm these theories.

To end up, we developed HelixDiff, a score-based diffusion network method for generating all-atom α-helix structures and enabling conditional peptide design matching hotspots for binding design. The effectiveness of direct conditional design is a crucial feature of HelixDiff, leveraging stochastic differential equations for smoother data-to-noise transformations and a more precise synthesis through reverse diffusion. This study highlights the capability of score-based generative diffusion models, such as HelixDiff, as peptide design tools, demonstrating their capacity to outperform classic GAN-based approaches.

## Conclusions

Our findings illustrate the benefits of using a score-based generative diffusion model, including improved sample generation quality, a valuable conditional generation pipeline, and improved stability in constructing D-peptide analogues. Future work includes implementing an algorithm to conditionally design novel bioactive peptides without masking the receptor in protein–peptide complexes.

## Experimental Section

### HelixDiff Model

Score-based generative modeling (SBGM) was initially introduced by Song et al.^30^ We also utilized it for HelixDiff. In the forward or sampling process, a random variable x_t is generated by simulating the stochastic differential equation (SDE) over time, commencing from an initial value of x0. This initial value, x0, undergoes perturbation through the addition of Gaussian noise during the forward process, yielding perturbed samples x_t. The forward process within the context of SBGM can be briefly characterized by the SDE
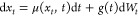
where *x*_*t*_ is the perturbed sample at time *t*, μ(*x*_*t*_, *t*) is the drift coefficient, *g*(*t*) is the diffusion coefficient, and *W*_*t*_ is a standard Wiener process that represents the random fluctuations in the system.

The backward process pertains to the generation of a sequence of purified samples in a reverse sequence conditioned on a target observation sequence. This reverse process is derived from the forward process through the utilization of gradients of the log-likelihood concerning the model parameters, often termed the score function. When provided with a forward stochastic differential equation (SDE), an analogous SDE in reverse time can be formulated as
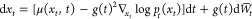
where ∇*x*__*t*__ log *p*_*t*_(*x*_*t*_) is the score function, σ(*t*) is the diffusion coefficient, and *W̅*_*t*_ is the standard Wiener process.

The score function measures how the log-likelihood of the perturbed sample, denoted as *x*_*t*_, evolves throughout the diffusion process. By calculating this score function, we can ascertain both the direction and magnitude of the gradient with respect to *x*_*t*_, which subsequently influences the drift term in the reverse-time stochastic differential equation (SDE). Typically, this score function is estimated using a neural network. Our study employed a UNet-based architecture featuring an attention module to estimate this score.

The variance exploding stochastic differential equation (VESDE) represents a novel type of SDE introduced as a more efficient alternative to conventional SDEs within score-based generative models.^30^ The primary objective of this diffusion process is to augment the noise variance within the SDE to prevent the sample from collapsing into a lower-dimensional subspace. Notably, the VESDE differs from the original SDE by having a tractable reverse process in which the drift coefficient of the forward process does not exert an influence. The forward process of VESDE is defined as
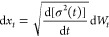
To generate samples from the reverse-time process, we employed a numerical solution approach to solve the reverse-time stochastic differential equation (SDE). Specifically, we utilized a predictor-corrector solver method. This method involves a sequential process where a predictor, such as the Euler–Maruyama method,^31^ is initially applied to estimate the state for the next time step. Subsequently, a corrector method, like Langevin Markov Chain Monte Carlo (MCMC),^32^ is employed to adjust the predicted state, ensuring that it adheres to the desired marginal distribution:
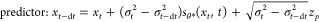

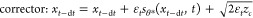


### Rosetta Energy Minimization

We employed the Rosetta relaxation protocol on both the generated and training data sets to validate the generated structures to yield low-energy full-atom structures. Employing Rosetta FastRelax as the primary tool, we explored various configurations, encompassing scenarios with no constraints, constraints limited to the main chain, and constraints extended to all atoms. Each configuration was iteratively executed across five independent trajectories for the generated and training data sets. Subsequently, the resulting structures were evaluated with the ref2015 energy function.^39^

### Design Strategy to Generate the D-GLP-1 Agonist

The query process for each helix segment was run independently using the trained HelixDiff model with hotspot-specific conditional generations module.^23^ The hotspot information, specifying the amino acid type and their positions, was provided to the model to generate full-atom helices containing these hotspots. Subsequently, a mirror transformation was applied to convert them into D-peptides, and a comparison of partial rmsd values relating to all of the hotspots was conducted. The selection criteria for each query involved identifying the lowest rmsd among the 200 hotspot-specific generations. GLP-1 hotspot residues were selected based on previous experimental data using as a starting structure the GLP-1R in complex with GLP-1 (5vai). HelixDiff was used to generate specific D-peptide helices based on a given set of GLP-1’s hotspots. The best-generated helices for each helix were assembled into D-peptide analogues over the GLP-1 receptor structure using Chimera.^33^

### Molecular Dynamics Simulations

All the initial structures and topology files for the MD simulations of the GLP-1 receptor (GLP-1R) in complex with different peptides embedded into a POPC:PSM (1:1) bilayer were built using the membrane builder generator implemented in the CHARMM-GUI web server.^34^ The GROMACS software package^35^ version 2019.3 was used to perform the molecular dynamics (MD) simulations of the GLP-1R+peptide complexes using the CHARMM36-m force field^36^ and the TIP3P water model.^37^ Two consecutive energy minimization (EM) schemes were used to relax the systems initially. The systems were then equilibrated in two sequential NVT ensemble simulations before being equilibrated in five successive NPT ensemble simulations at *p* = 1 bar and *T* = 310 K. We gradually released the position restraints applied to the protein-heavy atoms in both steps. Finally, the production NPT runs were performed for 300 ns for each system.

### Peptide Synthesis

All peptides were synthesized, purified, and characterized by Lifetein LLC. The purity of all peptides was higher than 95%. In the Supporting Information, we provide the details about the characterization of these peptides (molecular weight, purity, HPLC, and MS) (Figures S9 and 10 and Table S1).

### Circular Dichroism (CD) Analysis

Peptide secondary structure determination was carried out using a Jasco J-720 spectropolarimeter. Lyophilized peptide powders were dissolved in acetonitrile:PBS (1:2), and CD spectra were read immediately. Peptide concentrations were 20 μM for L-GLP-1 and 100 μM for D-GLP-1 analogues in acetonitrile:PBS (1:2). Samples were read using a 0.1 cm cuvette path length with ten accumulations per run and 50 nm/min scanning speed. All spectra were background subtracted.

### Cell Lines and Reagents

The HEK293 cell line was obtained from the American Type Culture Collection (ATCC). HEK293 cells were maintained in DMEM (Sigma) supplemented with 10% FBS and 1% penicilin/streptomycin/glutamine and the appropriate selection antibiotics when required.

### HTRF cAMP Assay

cAMP accumulation was measured using a HTRF cAMP Gs Dynamic kit (Cisbio Bioassays, 62AM4PEB) according to the manufacturer’s instructions. Briefly, HEK293 cells expressing hGLP-1R were trypsinized from subconfluent culture and seeded in a 96-well low-volume plate at a density of 2000 cells per well. Cells were treated with different concentrations of the L-GLP-1 peptide or D-GLP-1 peptides. After 4 h of incubation at 37 °C, cAMP d2 reagent and cAMP Eu-Cryptate antibodies were added to each well. After incubation at room temperature for 30 min in the dark, the plate was read using a Synergy 2 plate reader (BioTek) with excitation at 330 nm and emission at 620 and 665 nm. Data were used to calculate the EC_50_ value by fitting it to a nonlinear sigmoidal curve using GraphPad Prism 8.

### Western Blot

After cell starvation (0% FBS, 6 h), HEK293 cells expressing hGLP-1R were treated with 10 μM of L- or D-GLP-1 peptides for 4 h for AKT pathway activation and 36 h for GFP expression. Cells were lysed with lysis buffer (10 mM Tris-HCl pH 7.4, 1% SDS, 100 mM NaCl, 1 mM EDTA, 1× protease inhibitor mixture (Sigma)) for 30 min at 4 °C. Protein samples were separated on an SDS-PAGE gel and transferred to PVDF membranes. Transferred samples were immunoblotted with primary antibodies, followed by incubation with horseradish-peroxidase-conjugated secondary antibodies (Cell Signaling) and detected using enhanced chemiluminescence (Invitrogen). For quantification, band intensities were quantified using ImageJ software^38^ and normalized to the β-actin loading control values. Relative band intensity was presented as a ratio compared to the value of the L-GLP-1.

### Protease Stability Assays

For Proteinase K (Bioshop) assay, 50 μM of peptide was diluted in 80 μL of reaction buffer (10 mM Tris-HCl, 10 mM NaCl, pH 7.4, 5 μM CaCl_2_), and 12 μL was removed for the untreated T0 sample. Proteinase K was then added to a final concentration of 100 μg/mL concentration. Samples were incubated at 37 °C, 30 μL was removed after each time point, and protease activity was blocked by the addition of 10 mM PMSF. Protease-inactivated samples were frozen at −20 °C until further use. Frozen samples were analyzed by SDS-PAGE. Gels were stained by using Coomassie Brilliant Blue dye. The densitometry of bands was determined using ImageJ software.^38^ All samples were normalized to their respective untreated sample (T0).

Also, for the DPP-IV (ACROBiosystems) cleavage assay, 50 μM peptides was diluted in 80 μL of reaction buffer (100 mM Tris, pH 8.0), and 12 μL was removed for the untreated T0 sample. DPP-IV was then added to a final concentration of 50 μg/mL. Further samples were prepared and analyzed using the above procedure.

### Statistical Analysis

Statistical significance was analyzed by a two-tailed unpaired Student’s *t* test using MS Excel. A *P* value less than 0.05 was considered statistically significant.

## Data Availability

The source code and data sets are available at github (https://github.com/xxiexuezhi/HelixDiff).

## Supporting Information Available

Sequences of the peptides evaluated experimentally in this study, graphical scheme of the features encoded in HelixDiff to generate novel helices, sequence identity of the α-L-helix structures unconditionally generated using HelixDiff and HelixGAN with the training set, structural superposition of the designed helices generated with HelixDiff and PEP-FOLD4, *de**novo* design of α-L-helical peptides with constrained hotspots using the hotspot-specific generation method implemented in HelixDiff, *de**novo* design of α-D-helical peptides with constrained hotspot residues using the hotspot-specific generation method implemented in HelixDiff, rmsd distribution profiles of the best-generated structures for each D-GLP-1 helix fragment using HelixDiff, activity profile of L-GLP-1 and the D-GLP-1 peptides over HEK293 cells stably expressing GLP-2R and CRE-luciferase, resistance of the designed D-GLP-1 analogues to Proteinase K and DPP-IV degradation, analytical characterization of the D-GLP-1_diff_Acetylated peptide provided by the Lifetein company, and analytical characterization of the L-GLP-1 peptide provided by the Lifetein company (PDF)
Transparent Peer Review report available (PDF)

## Abbreviations

AKT: protein kinase B
cAMP: cyclic adenosine monophosphate
CD: circular dichroism
DMEM: Dulbecco’s Modified Eagle’s Medium
DMSO: dimethyl sulfoxide
D-GLP-1: glucagon-like peptide 1 D-peptide analogue
DPP-IV: dipeptidyl peptidase 4
EM: energy minimization
GLP-1: glucagon-like peptide 1
GLP-2: glucagon-like peptide 2
GLP-1R: glucagon-like peptide 1 receptor
GLP-2R: glucagon-like peptide 2 receptor
GPCR-Gs: G protein-coupled receptors
HPLC: high pressure liquid chromatography
HTRF: homogeneous time resolved fluorescence
EC_50_: half maximal effective concentration
L-GLP-1: glucagon-like peptide 1
MD: molecular dynamics
MS: mass spectra
PBS: phosphate-buffered saline
PDB: protein data bank
PKA: protein kinase A
PMSF: phenylmethylsulfonyl fluoride
PSM: palmitoylsphingomyelin
POPC: 1-palmitoyl-2-oleoylphosphatidylcholine, palmitoyloleoylphosphatidylcholine
ProtK: proteinase K
RI: retro-inverse
rmsd: root-mean-square deviation
RMSF: root-mean-square fluctuation
