# Supplementary material for: HelixDiff, a Score-Based Diffusion Model for Generating All-Atom α-Helical Structures
Source: ACS Cent Sci. 2024 Apr 5;10(5):1001–11. doi: 10.1021/acscentsci.3c01488 (PMC11117309; doi:10.1021/acscentsci.3c01488)
Supplement: Supplementary file 1 — oc3c01488_si_001.pdf [file oc3c01488_si_001.pdf]

Supporting Information

HelixDiff a Score-based Diffusion Model for Generating All-atom Alpha Helical structures

Xuezhi Xie [1,2], Pedro A Valiente[1], Jisun Kim[1], and Philip M Kim[1, 2, 3*]

[1] Donnelly Centre for Cellular and Biomolecular Research, University of Toronto, Toronto, ON M5S 3E1, Canada.
[2] Department of Computer Science, University of Toronto, Toronto, ON M5S 3E1, Canada.
[3] Department of Molecular Genetics, University of Toronto, Toronto, ON M5S 3E1, Canada.

Corresponding author

*email: pm.kim@utoronto.ca

Contents:

Table S1 Sequences of the peptides evaluated experimentally in this study.

Figure S1 Graphical scheme of the features encoded in HelixDiff to generate novel helices. Helices are encoded as the combined vectors of the primary sequence information using one-hot encoding and structural information like bonds, phi, psi, omega, and chi angles. The structural information is further divided into the main chain and side chain. The main chain is represented as phi (ϕ), psi (ψ), omega (ω), and bond angles, while the side chains are defined using the five chi (χ) angles.

Figure S2 Sequence identity of the alpha L-helices structures unconditionally generated using HelixDiff and HelixGAN with the training set. A) HelixDiff. **B)** HelixGAN. Two thousand alpha helices were randomly selected and compared to the nearest natural helices from the training data to calculate sequence identity for each algorithm. Both models were encoded similarly and trained with identical datasets to ensure a fair and unbiased comparison.

Figure S3. Structural superposition of the designed helices generated with HelixDiff and PEP-FOLD4. The helices with HelixDiff are emphasized in gray, while the ones generated by PEP-FOLD4 are presented in a light orange.

Figure S4 De *novo* design of alpha L-helical peptides with constrained hotspots using the hotspots-specific generation method implemented in HelixDiff. A) Assessment of the performance of HelixDiff to generate alpha L-helical peptides with constrained hotspots in a test set of 90 samples where 3 random hotspots were selected. The RMSD was calculated between the chosen hotspot atoms in each generated and target helix. **B)** RMSD of the HelixGAN's performance in a similar test set. **C)** RMSD distribution of the generated alpha-helical structures using HelixDiff and HelixGAN. **D)** Rosetta scores distribution of the generated alpha-helical structures using HelixDiff and HelixGAN.

Figure S5 De *novo* design of alpha D-helical peptides with constrained hotspot residues using the hotspots-specific generation method implemented in HelixDiff. A) Assessment of the performance of HelixDiff to generate alpha D-helical peptides with constrained hotspot

residues in a test set of 90 samples where three random hotspots were selected. The RMSD was calculated between the chosen hotspot atoms in each generated and target helix. **B)** RMSD of the HelixGAN's performance in a similar test set. **C)** RMSD distribution of the generated alpha-helical structures using HelixDiff and HelixGAN. **D)** Rosetta scores distribution of the generated alpha-helical structures using HelixDiff and HelixGAN. **E)** Assessment of the performance of HelixDiff to generate alpha D-helical peptides with constrained hotspots in a test set of 90 samples where four random hotspots were selected. **F)** Rosetta score distribution of the set of novel D-helices generated with HelixDiff considering four random hotspot residues.

Figure S6 RMSD distribution profiles of the best-generated structures for each D-GLP-1 helix fragment using HelixDiff. A) For helix1 were selected H7, E9, and F12 as hotspots in GLP-1. **B)** For helix2 were selected T13, D15, and Y19 as hotspots in GLP-1. **C)** For helix3 were selected F28, I29, and L32 as hotspots in GLP-1. All peptides were generated with constrained hotspots using the hotspot-specific inpainting method implemented in HelixDiff. We did 1920 (batch size 64, 30 rounds) samples for each helix.

Figure S7 Activity profile of L-GLP-1 and the D-GLP-1 peptides over HEK293 cells stably expressing GLP-2R and CRE-luciferase.

Figure S8 The designed D-GLP-1 analogs are resistant to Proteinase K and DPP-IV degradation. A) SDS-PAGE images of the designed D-peptides and L-GLP-1 treated with Proteinase K in 30 min intervals. **B)** SDS-PAGE images of the designed D-peptides treated with DPP-IV in 30 min intervals. Gels were stained with Coomassie Brilliant Blue dye.

Figure S9 Analytical characterization of the D-GLP-1_diff_Acetylated peptide provided by the Lifetein company. A) HPLC report. **B)** Mass spectrometry report.

Figure S10 Analytical characterization of the L-GLP-1 peptide provided by the Lifetein company. A) HPLC report. **B)** Mass spectrometry report.

Table S1 Sequences of the peptides evaluated experimentally in this study

Peptides	Full sequence[a]	Purity (%)	MW (g/mol)
**L-GLP1**	**H**A**E**GT**FT**S**D**VSS**Y**LEGQAAKE**FI**AW**L**VKGRG	96.80	3355.80
**D-GLP-1_diff_Acetylated**	Acetyl-**HEA**S**TFADS**AA**Y**TNAKAAA**IF**WA**L**ARLL	97.20	3023.55
**D-GLP-1_mirror_image_search**	RGKAF**L**EL**FI**LAAGELIEAI**D**R**TF**TG**E**A**H**	95.26	3298.69

[a]The full sequence of **D-GLP-1_diff_Acetylated** is obtained by combining helix1+helix2+helix3. The hotspots and matched residues in L-GLP-1 and the D-peptides are highlighted in bold. The hotspot residues selected to design both peptides are highlighted in yellow. Y19, which was only chosen as a hotspot to design D-GLP-1_diff_Acetylated, is highlighted in green.

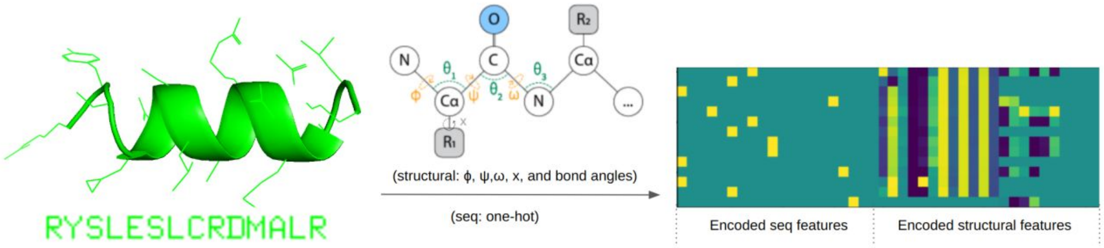

Figure S1 Graphical scheme of the features encoded in HelixDiff to generate novel helices. Helices are encoded as the combined vectors of the primary sequence information using one-hot encoding and structural information like bonds, phi, psi, omega, and chi angles. The structural information is further divided into the main chain and side chain. The main chain is represented as phi (ϕ), psi (ψ), omega (ω), and bond angles, while the side chains are defined using the five chi (χ) angles.

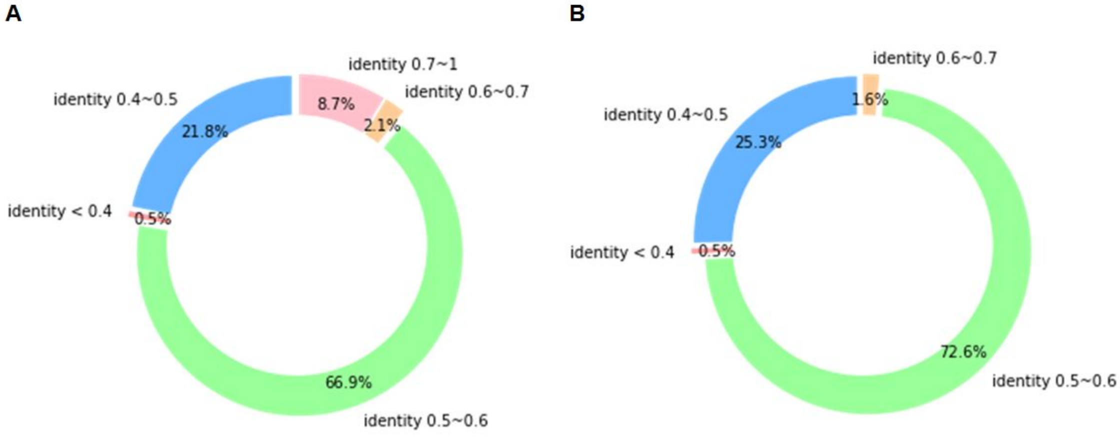

Figure S2 Sequence identity of the alpha L-helices structures unconditionally generated using HelixDiff and HelixGAN with the training set. A) HelixDiff. B) HelixGAN. Two thousand alpha helices were randomly selected and compared to the nearest natural helices from the training data to calculate sequence identity for each algorithm. Both models were encoded similarly and trained with identical datasets to ensure a fair and unbiased comparison

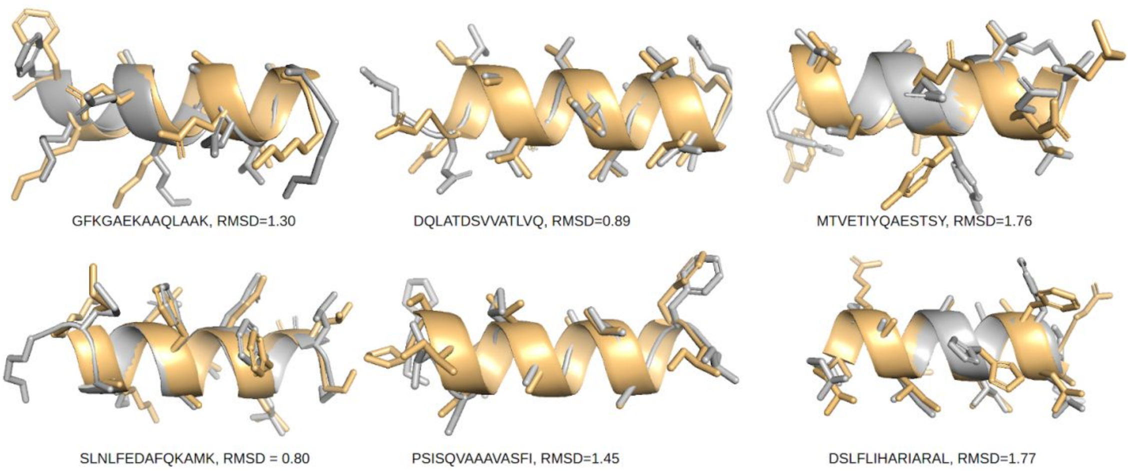

Figure S3. Structural superposition of the designed helices generated with HelixDiff and PEP-FOLD4. The helices with HelixDiff are emphasized in gray, while the ones generated by PEP-FOLD4 are presented in a light orange.

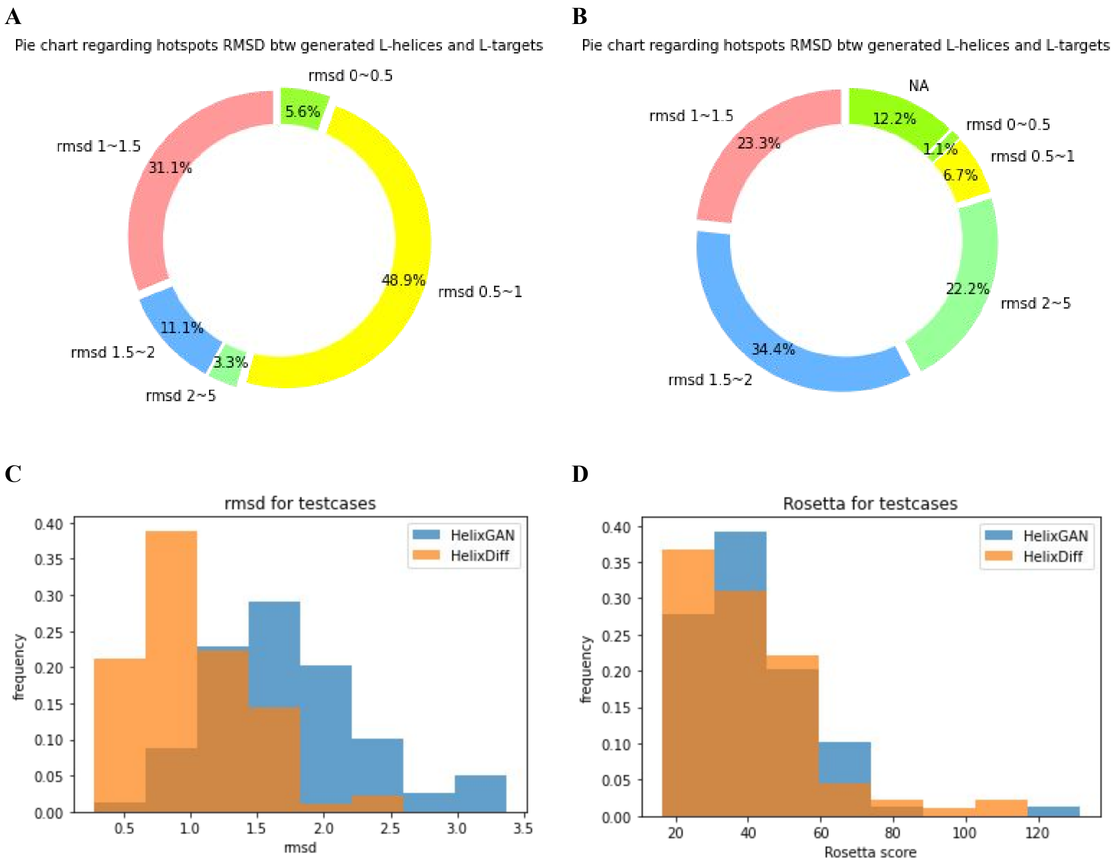

Figure S4 De *novo* design of alpha L-helical peptides with constrained hotspots using the hotspots-specific generation method implemented in HelixDiff. A) Assessment of the performance of HelixDiff to generate alpha L-helical peptides with constrained hotspots in a test set of 90 samples where 3 random hotspots were selected. The RMSD was calculated between the chosen hotspot atoms in each generated and target helix. **B)** RMSD of the HelixGAN's performance in a similar test set. **C)** RMSD distribution of the generated alpha-helical structures using HelixDiff and HelixGAN. **D)** Rosetta scores distribution of the

generated alpha-helical structures using HelixDiff and HelixGAN.

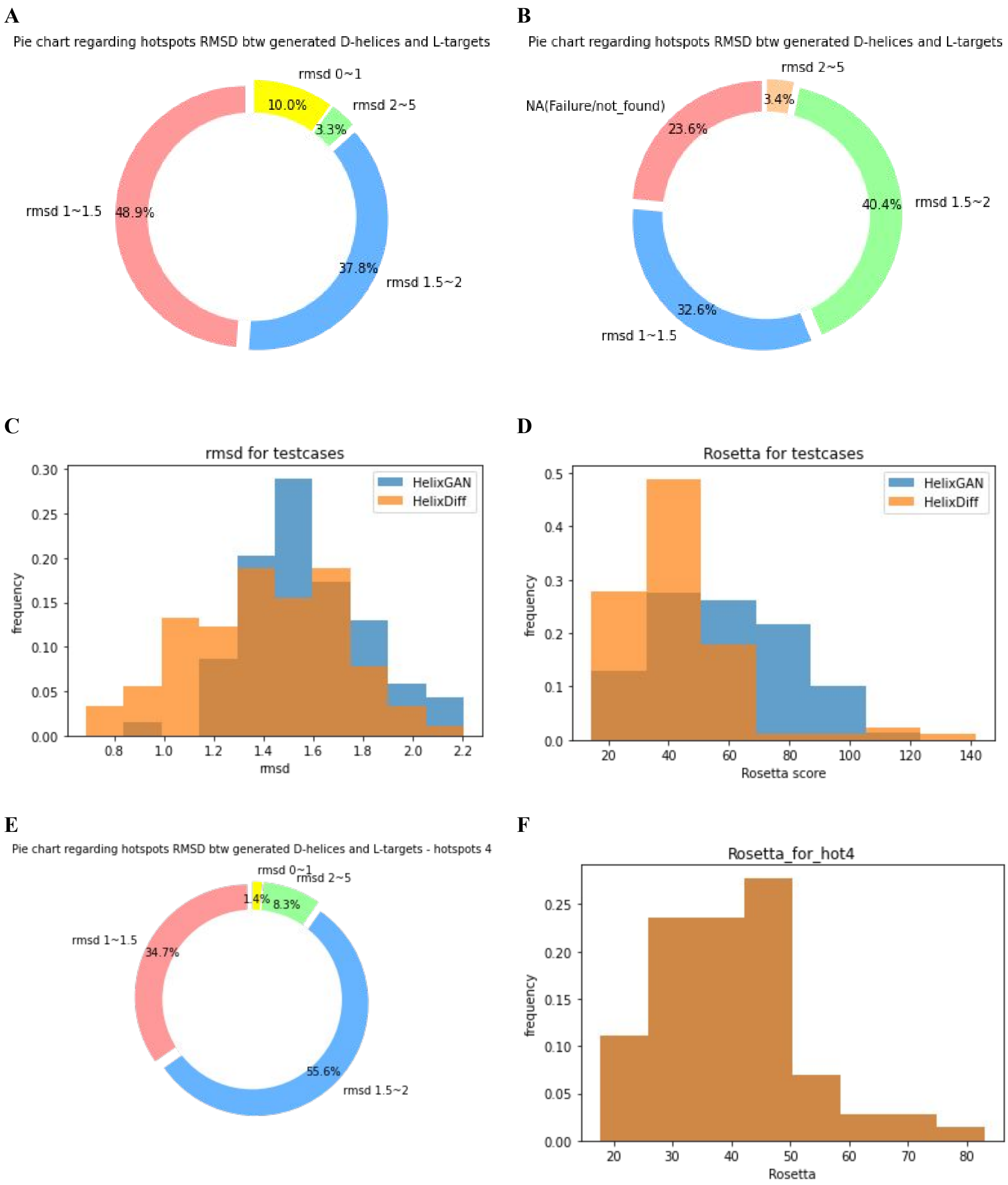

Figure S5 De *novo* design of alpha D-helical peptides with constrained hotspot residues using the hotspots-specific generation method implemented in HelixDiff. A) Assessment of the performance of HelixDiff to generate alpha D-helical peptides with constrained hotspot residues in a test set of 90 samples where three random hotspots were selected. The RMSD was calculated between the chosen hotspot atoms in each generated and target helix. **B)** RMSD of the HelixGAN's performance in a similar test set. **C)** RMSD distribution of the generated alpha-helical structures using HelixDiff and HelixGAN. **D)** Rosetta scores distribution of the generated alpha-helical structures using HelixDiff and HelixGAN. **E)** Assessment of the performance of HelixDiff to generate alpha D-helical peptides with

constrained hotspots in a test set of 90 samples where four random hotspots were selected. **F)** Rosetta score distribution of the set of novel D-helices generated with HelixDiff considering four random hotspot residues.

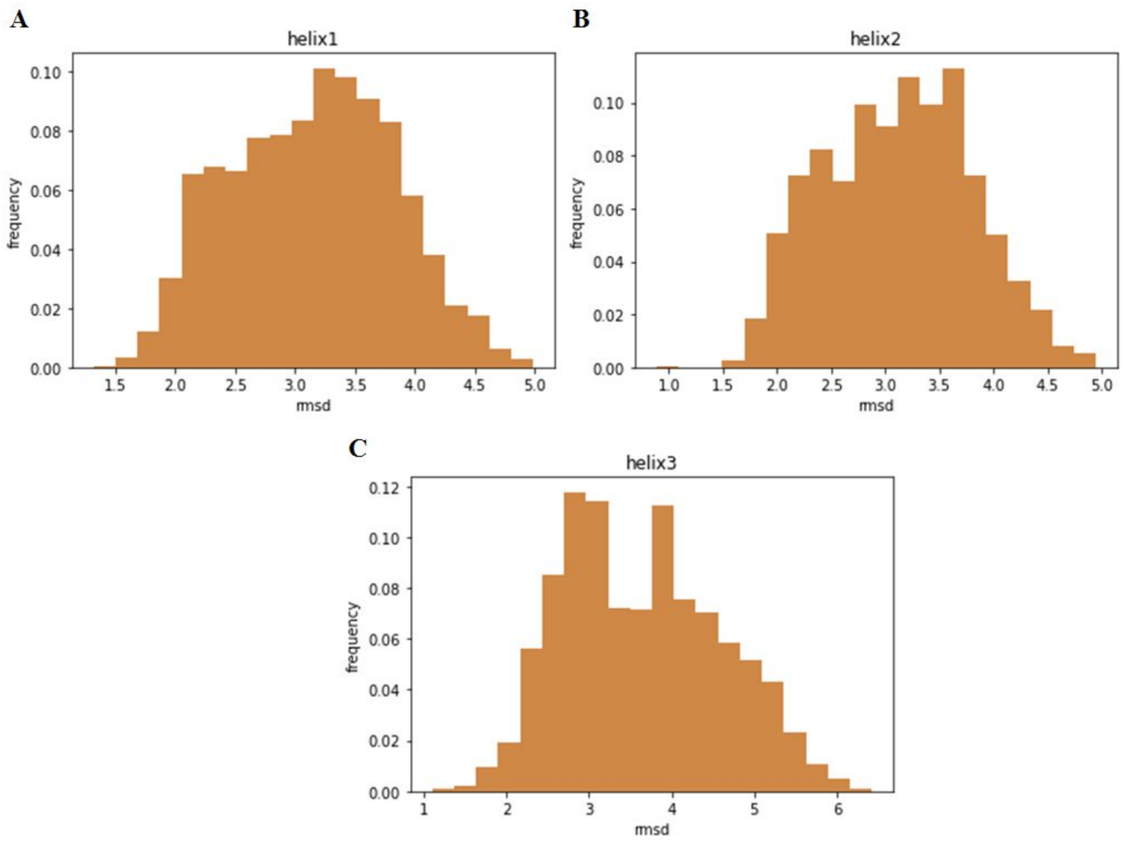

Figure S6 RMSD distribution profiles of the best-generated structures for each D-GLP-1 helix fragment using HelixDiff. A) For helix1 were selected H7, E9, and F12 as hotspots in GLP-1. **B)** For helix2 were selected T13, D15, and Y19 as hotspots in GLP-1. **C)** For helix3 were selected F28, I29, and L32 as hotspots in GLP-1. All peptides were generated with constrained hotspots using the hotspot-specific inpainting method implemented in HelixDiff. We did 1920 (batch size 64, 30 rounds) samples for each helix.

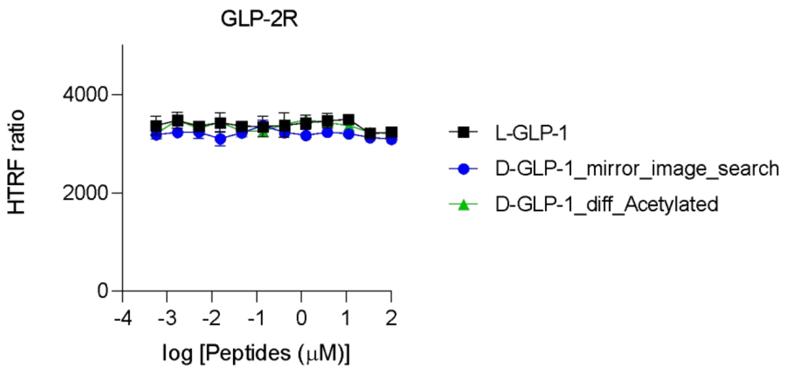

Figure S7 Activity profile of L-GLP-1 and the D-GLP-1 peptides over HEK293 cells stably expressing GLP-2R and CRE-luciferase.

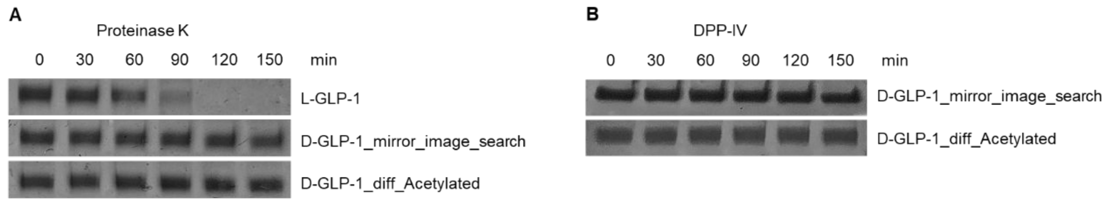

Figure S8 The designed D-GLP-1 analogs are resistant to Proteinase K and DPP-IV degradation. A) SDS-PAGE images of the designed D-peptides and L-GLP-1 treated with Proteinase K in 30 min intervals. **B)** SDS-PAGE images of the designed D-peptides treated with DPP-IV in 30 min intervals. Gels were stained with Coomassie Brilliant Blue dye.

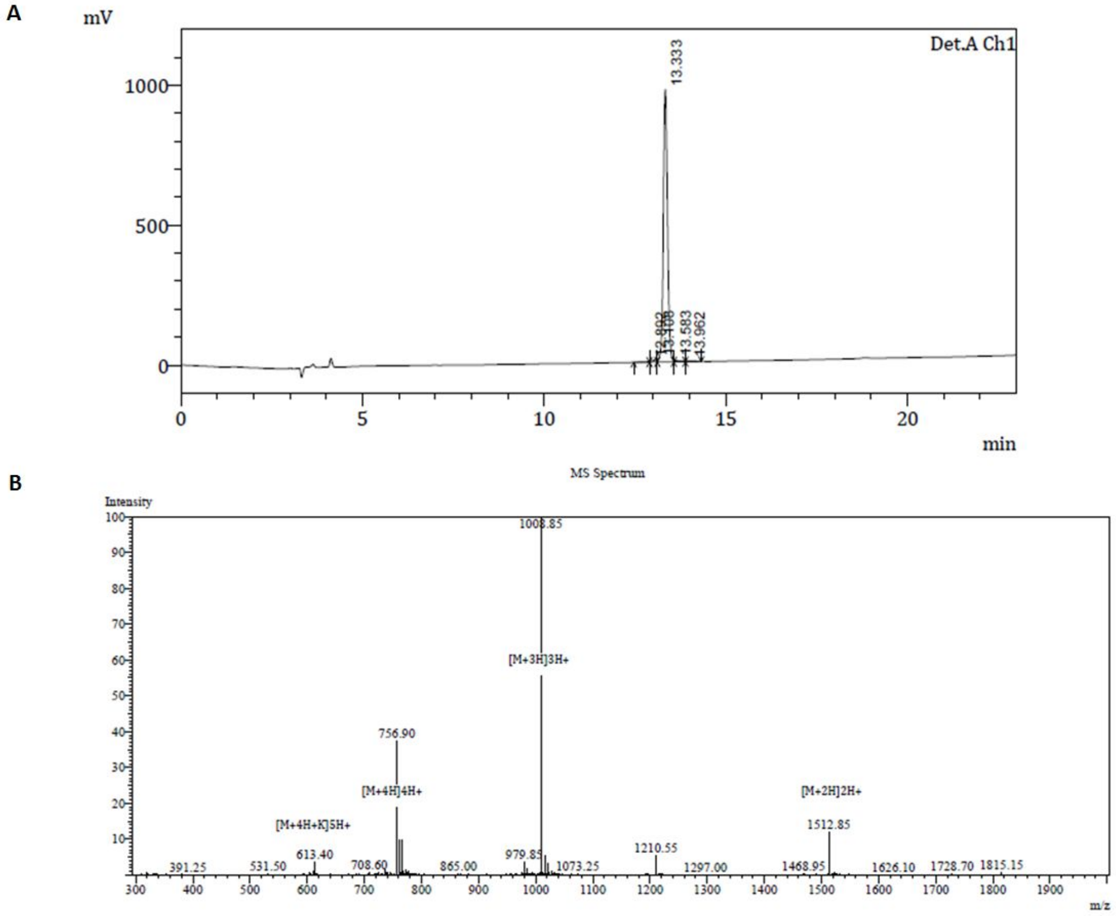

Figure S9 Analytical characterization of the D-GLP-1_diff_Acetylated peptide provided by the Lifetein company. A) HPLC report. **B)** Mass spectrometry report.

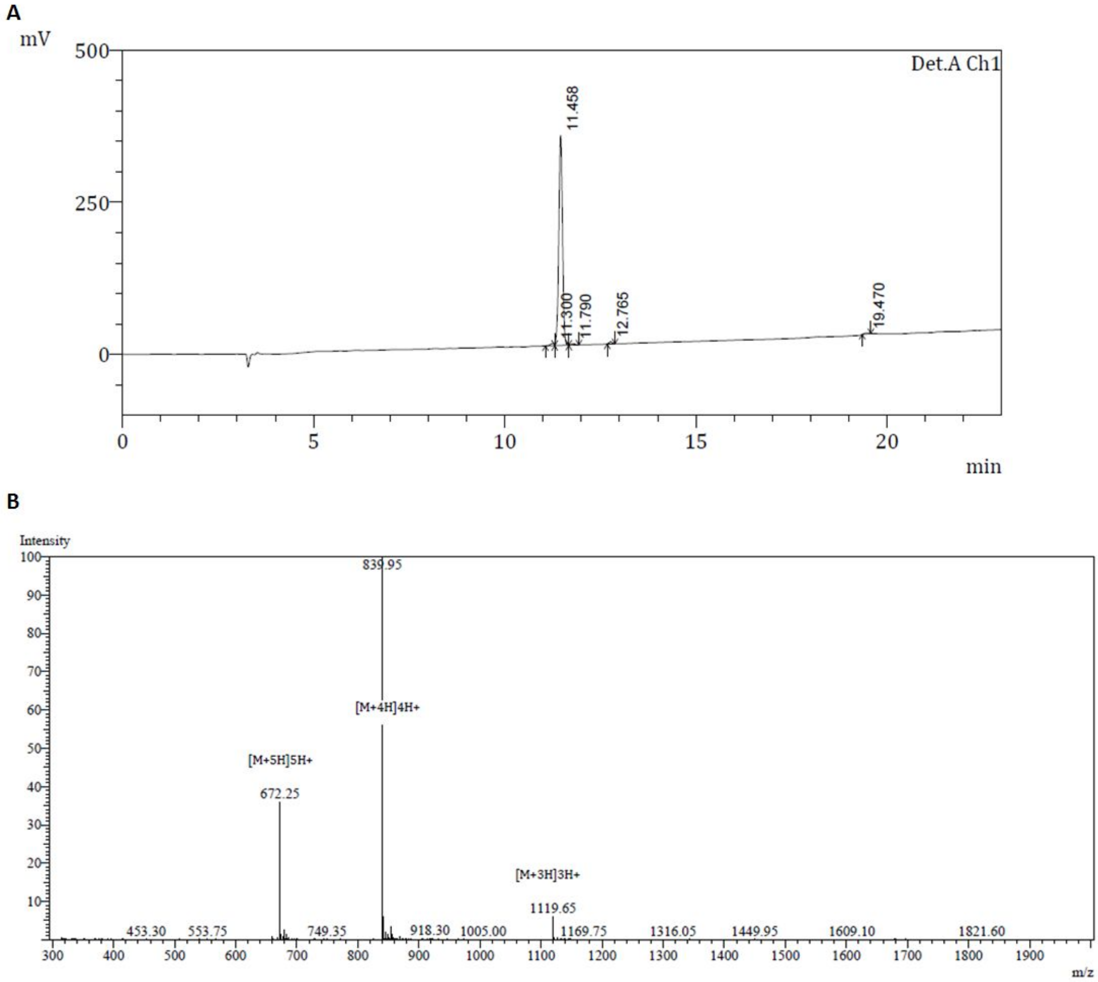

Figure S10 Analytical characterization of the L-GLP-1 peptide provided by the Lifetein company. A) HPLC report. **B)** Mass spectrometry report.
