# Supplementary material for: HelixDiff, a Score-Based Diffusion Model for Generating All-Atom α-Helical Structures
Source: ACS Cent Sci. 2024 Apr 5;10(5):1001–11. doi: 10.1021/acscentsci.3c01488 (PMC11117309; doi:10.1021/acscentsci.3c01488)
Supplement: Supplementary file 2 — oc3c01488_si_002.pdf [file oc3c01488_si_002.pdf]

oc-2023-01488a.R1

Name: Peer Review Information for "HelixDiff a Score-based Diffusion Model for Generating All-atom Alpha Helical Structures"

First Round of Reviewer Comments

Reviewer: 1

Comments to the Author

The manuscript describes a diffusion method to generate helical peptides, HelixDiff, which uses one-hot encoded sequence and torsional descriptions of amino acid conformations to produce a full-atom model. The authors used the model to design a helical peptide in the D-amino acid space to bind to GLP1R and found that the design peptide led to the activation of the receptor, by probing a few markers with western blots. The stability of the designed peptide is also assessed to be stable. The results showing GLP1R activation and the ability to custom design stable D-peptides are very interesting.

The authors offered a number of different experiments to assess the model's performance, both in silico and in vitro, but there are some clear gaps/issues concerning the experiments. The manuscript can be improved with following suggestions:

Major:

1) Fig1B and 1C seem to suggest that the peptides are spatially constrained to allow hotspot residues be maintaining their positions in the red boxes, but on further reading, it seems to only use the hotspots' positions in the sequence and amino acid identity. If this point can be clarified, it would be very helpful. The confusion is that the process is referred to as "inpainting" but normally impaintings imply 2D contact interactions, which does not seem to be the case here.

2) The proposed Rosetta score comparison is problematic. (Fig. 2 and S4) Rosetta scores reported were positive in value, and that is usually not considered viable designs. The implication is that the generated structures either have internal clashes or has distortion in bond-geometries. Generative models don't always honor molecular forcefield parameters, so this kind of behavior is expected. However, to illustrate validity by Rosetta, the authors might want to try minimizing the generated structures using the Rosetta forcefield (with/without coordinate constraints: perhaps following

https://www.rosettacommons.org/docs/latest/rosetta_basics/preparation/preparing-structures#how-do-i-prepare-structures) and see if the structures change significantly. This is important because the structure representation (Fig. S1) seems to suggest that mainly internal coordinates (angles) were used, it is not clear how the accurately the model can maintain chemical structures of amino acids.

3) Conditional generation of one-hot-encoded hotspots is quite interesting, but as implemented, it seems to require a template structure/sequence to create the D-peptide mimetic. In this fashion, the manuscript seems to be missing a very critical control, which is a simple, generic helix simply hosting the hotspot residues. HelixDiff model does not seem to use any information from the target protein and fully depends on the choice of hotspot residues. While the manuscript offered very nice controls using L-GLP-1 and D-GLP-1_mirror_image_search from a prior method, The observation that the mirror_image peptide showing similar activation in the HTRF experiment potentially suggest a lower bound background when matching hotspot residues are present. To address this possibility and to highlight that a design process is required, a critical control is perhaps a poly alanine carrying the same hotspots (may need some polar residues for solubility) or a peptide with the same composition but scrambled sequence, still with the same and/or shuffled hotspots. Because the HelixDiff is made to mimic and stabilize the hotspot residues, what if having the hotspots alone without design could already achieve the same results?

A few minor suggestions:

1) in FigS2, the colors for % range don't seem to match; it would be easier to compare if they are the same.

2) There is a typo on Page 7. The last mention of helix2 should be helix 3.

3) Figure 3 has no legend for H. Also it is not clear where licorice color is shown, just labels?

Author's Response to Peer Review Comments:

Reviewer: 1
Comments to the Author

Reviewer: 1

Recommendation: Publish in ACS Central Science after minor revisions noted.

Comments:
The manuscript describes a diffusion method to generate helical peptides, HelixDiff, which uses one-hot encoded sequence and torsional descriptions of amino acid conformations to produce a full-atom model. The authors used the model to design a helical peptide in the D-amino acid space to bind to GLP1R and found that the design peptide led to the activation of the receptor, by probing a few markers with western blots. The stability of the designed peptide is also assessed to be stable. The results showing GLP1R activation and the ability to custom design stable D-peptides are very interesting.
The authors offered a number of different experiments to assess the model's performance, both *in silico* and in vitro, but there are some clear gaps/issues concerning the experiments. The manuscript can be improved with following suggestions:

Major:
1) Fig1B and 1C seem to suggest that the peptides are spatially constrained to allow hotspot residues be maintaining their positions in the red boxes, but on further reading, it seems to only use the hotspots' positions in the sequence and amino acid identity. If this point can be clarified, it would be very helpful. The confusion is that the process is referred to as "inpainting" but normally impaintings imply 2D contact interactions, which does not seem to be the case here.

We thank the valuable comment from the reviewer. We acknowledge the potential confusion regarding Figures 1B and 1C and the terminology used to describe the process. Our conditional generation approach is based exclusively on hotspot positions and amino acid types, resulting in superior conformation matching, especially in D-peptide design settings. The chi angles matrix regarding the side chains is not given for a better hotspot confirmation matching, especially for D-peptide design. We agree that the term "inpainting" could suggest 2D contact interactions, which is inaccurate in our methodology. In our approach, "inpainting" refers to the art of reconstructing missing parts of an image and can be extended to our case - sequence and angle matrix. In the revised version of the manuscript, we changed the phrase "*hotspots-specific inpainting modules*" to "*hotspots-specific generation modules*" to provide a more precise explanation and improve readers' understanding.

The paragraph on page 3,4 are revised as below:

“We next integrated a conditional hotspot-specific generation module tailored explicitly to the receptor of interest in Fig 1 B and C. We defined the hotspot residues as those that have a critical contribution to target recognition, binding, and receptor activation. We then constrained the structural generation of the novel peptides to a set of identified hotspots to produce functional designs. The hotspot residue information served as contextual cues for the module to reconstruct the remaining data. The conditional generation process focused on fulfilling the encoding matrix, resulting in more targeted and precise generations. We could generate a variety of realistic conformational rotamers based on hotspot residues and amino acid types, resulting in superior conformation matching, especially in D-peptide design settings.”

2) The proposed Rosetta score comparison is problematic. (Fig. 2 and S4) Rosetta scores reported were positive in value, and that is usually not considered viable designs. The implication is that the generated structures either have internal clashes or has distortion in bond-geometries. Generative models don't always honor molecular forcefield parameters, so this kind of behavior is expected. However, to illustrate validity by Rosetta, the authors might want to try minimizing the generated structures using the Rosetta force field (with/without coordinate constraints: perhaps following https://www.rosettacommons.org/docs/latest/rosetta_basics/preparation/preparing-structures#how-do-i-prepare-structures) and see if the structures change significantly. This is important because the structure representation (Fig. S1) seems to suggest that mainly internal coordinates (angles) were used, it is not clear how the accurately the model can maintain chemical structures of amino acids.

We thank the valuable comment from the reviewer. To address this concern and validate our results, we randomly selected 600 structures from both the generated and the training data (as a control group) for further investigation. We applied the Rosetta relaxation protocol with and without constraints for each sample. To provide a comprehensive comparison, we calculated both mainchain RMSD and full-atom RMSD, as side chain interactions are crucial in our study. Our analysis revealed that the RMSD distributions for the training and generated data were similar, with differences in median and average values of approximately 0.1–0.2 Å. Significantly, after energy minimization with the rosetta force field, the scores obtained for most structures were negative (Figure 1C-D, Figure 3 manuscript). This result suggests that our generated data exhibit comparable chemical properties to experimental data, bolstering the validity of our results. In the revised version of the manuscript, we presented the RMSD and Rosetta score distributions between the structures before and after relaxation as new Figure 3 and Table 1.

Table 1. RMSD of the generated and training structures after minimization with Rosetta relax

	RMSD relax for generated data (A°)		RMSD Relax for training data (A°)	
	no constraints	constraints	No constraints	constraints
Main chain (avg)[a]	0.89	0.82	0.77	0.70
Main chain (med)[b]	0.85	0.78	0.73	0.65
All atom (avg)[a]	1.82	1.72	1.70	1.59
All atom (med)[b]	1.78	1.68	1.65	1.56

We calculated the rmsd after the relaxation using the Rosetta relax protocol for each structure, considering the main chain and all atoms. [a] average RMSD of a set of structures. [b] median RMSD of a set of structures

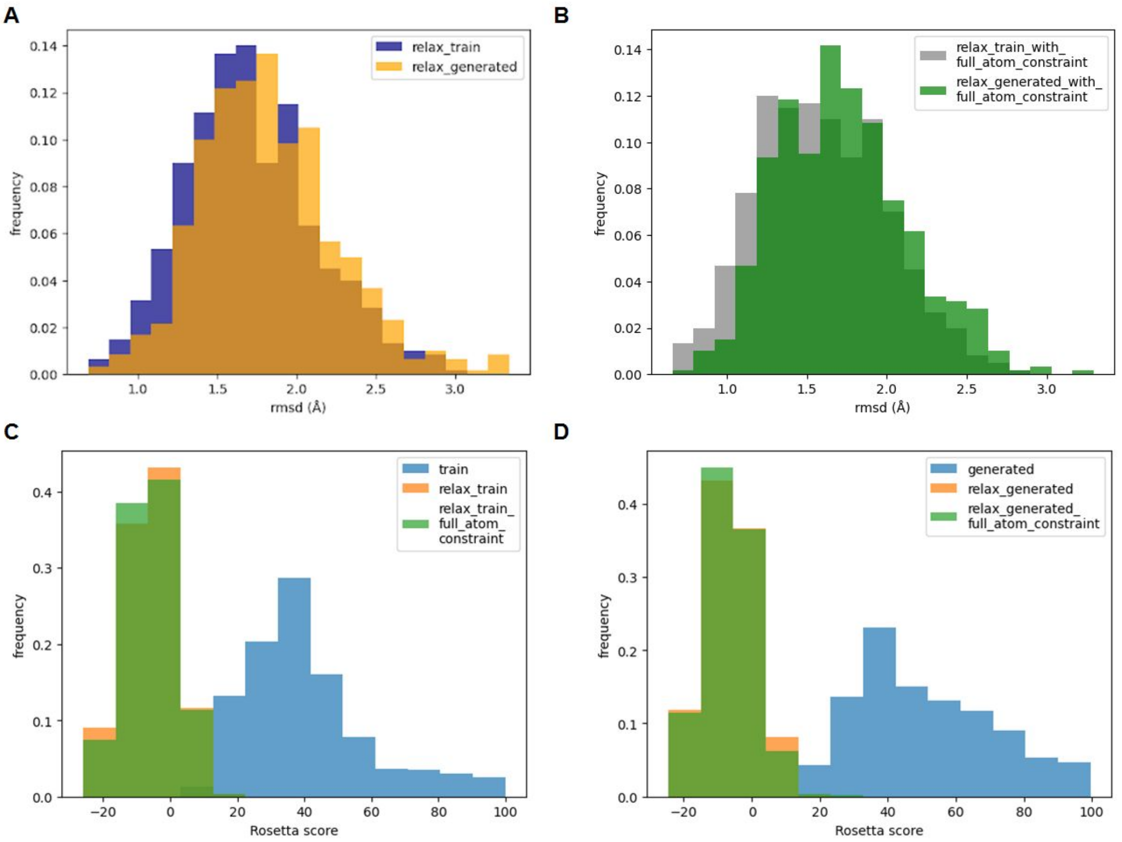

Figure 1 RMSD and rosetta score distributions of the generated structures before and after Rosetta relaxation. A) RMSD distribution of the structures in the training and generated data sets after relaxation without constraints. **B)** RMSD distribution of the structures in the training and generated data sets after relaxation using all-atom constraints. **C)** Rosetta scores distributions of the structures in the training set without relaxation (blue), after relaxation without constraints (orange), and after relaxation with constraints (green). **D)** Rosetta scores distributions of generated structures without relaxation (blue), after relaxation without constraints (orange), and after relaxation with constraints (green). We randomly collected 600 structures from the training and generated data. Following the rosetta relaxation protocol, we calculated the rmsd between the structures before and after relaxation. The rmsd values were calculated considering all the atoms in each structure.

3) Conditional generation of one-hot-encoded hotspots is quite interesting, but as implemented, it seems to require a template structure/sequence to create the D-peptide mimetic. In this fashion, the manuscript seems to be missing a very critical control, which is a simple, generic helix simply hosting the hotspot residues. HelixDiff model does not seem to use any information from the target protein and fully depends on the choice of hotspot residues. While the manuscript offered very nice controls using L-GLP-1 and D-GLP-1_mirror_image_search from a prior method, The observation that the mirror_image peptide showing similar activation in the HTRF experiment potentially suggest a lower bound background when matching hotspot residues are present. To address this possibility and to highlight that a design process is required, a critical control is perhaps a poly alanine carrying the same hotspots (may need some polar residues for solubility) or a peptide with the same composition but scrambled sequence, still with the same and/or shuffled hotspots.

Because the HelixDiff is made to mimic and stabilize the hotspot residues, what if having the hotspots alone without design could already achieve the same results?

We thank the valuable comment from the reviewer. We designed D-GLP-1_diff_Acetylated, a D-peptide agonist with the same binding mode orientation as GLP-1 using HelixDiff. To build this new D-peptide, we employed almost the same set of hotspot residues in GLP-1 that we used to create D-GLP-1_mirror_image_search, our earlier retro-inverted D-peptide design. The new D-peptide design activated the GLP-1 receptor signaling similarly to D-GLP-1_mirror_image_search. This finding indicates that matching critical hotspots for the GLP-1 function is more relevant to retaining the GLP-1's agonist effect than the sequence orientation of the D-peptide analog. To predict the impact over the peptide binding mode stability of mutating all the non-hotspots residues to alanine, we designed a polyalanine D-peptide carrying the same hotspots named D-GLP-1_diff_Acetylated_only_hotspots. As expected, the D-GLP-1 analog with all the non-hotspot residues mutated to alanine showed a more significant structural fluctuation than the wild-type D-peptide **(Figure 2, Figure 4 manuscript**). These mutations will make the peptide more hydrophobic and could hinder the peptide's solubility and functional activity. On the other hand, we previously showed that a scrambled form of the D-GLP-1_mirror_image_search peptide was inactive. Thus, we hypothesized that a scrambled variant of D-GLP-1_diff_Acetylated would be inactive, given that we designed both D-peptides using almost the same set of hotspots in the GLP-1 sequence. Further experiments will be needed in the future to confirm our predictions. We included this new data in the result (**Figure 4C and D, Table S1**) and discussion sections of the manuscript.

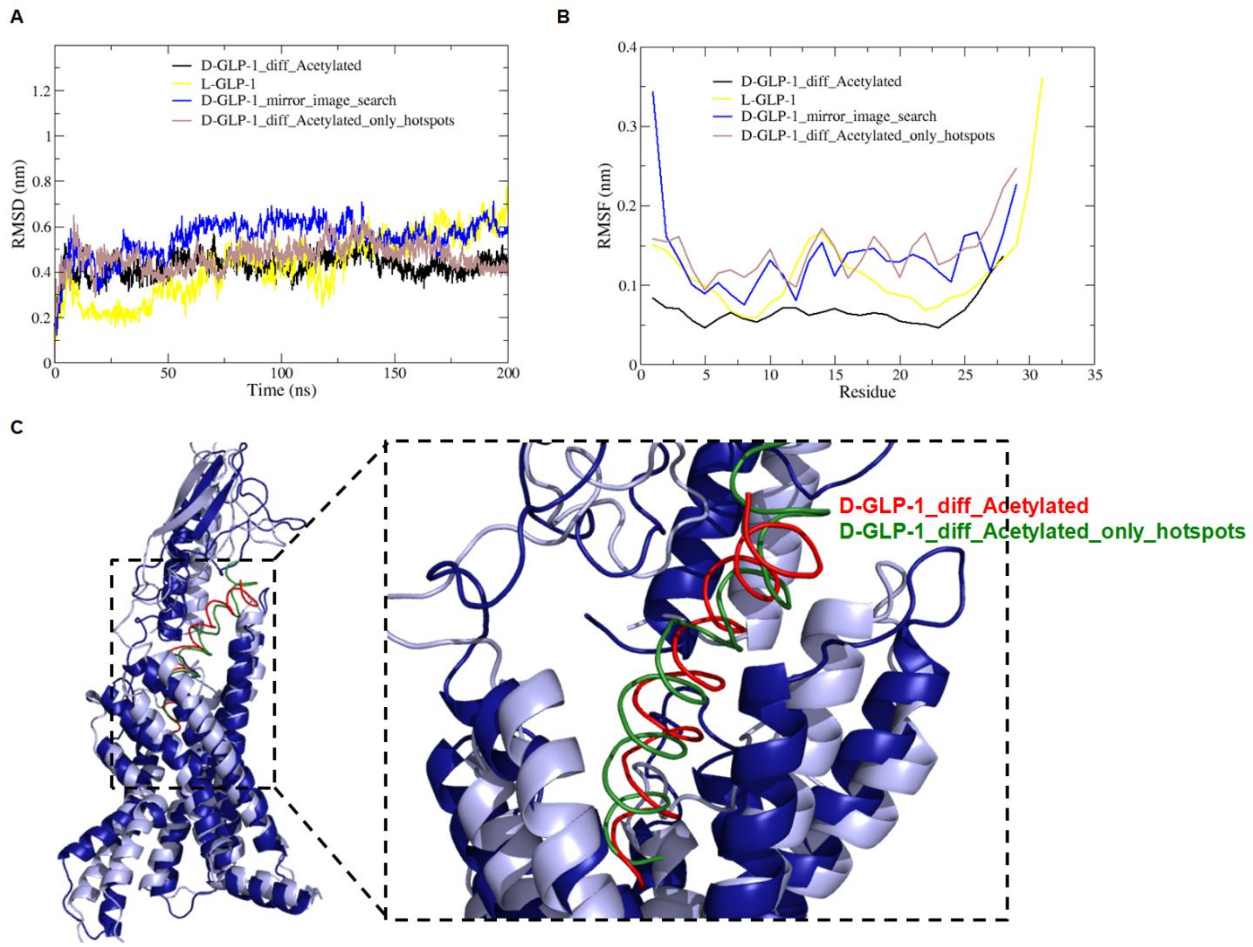

Figure 2 Impact of mutating the non-hotspot residues to alanine in the structural stability of D-GLP-1_diff_Acetylated along MD simulations. A) Root mean square deviation (RMSD) of the heavy atoms of L-GLP-1 and the D-GLP-1 analogs bound to the GLP-1R. **B)** Root mean square fluctuation (RMSF) per residue of the heavy atoms of L-GLP-1 and the D-GLP-1 analogs bound to the GLP-1R. **C)** Structural superposition of the most representative cluster extracted from the MD simulation of GLP-1R bound to D-GLP-1_diff_Acetylated and D-GLP-1_diff_Acetylated_only_hotspots. The D-GLP-1_diff_Acetylated analog is shown in red, while D-GLP-1_diff_Acetylated_only_hotspots is displayed in green. In dark blue is shown the GLP-1R coupled to D-GLP-1_diff_Acetylated, while in light blue is represented the receptor bound to D-GLP-1_diff_Acetylated_only_hotspots.

Table S1 Sequences of the peptides evaluated experimentally in this study

Peptides	Full sequences[a]
**L-GLP-1**	**H**A**E**GT**FT**S**D**VSS**Y**LEGQAAKE**FI**AW**L**VKGRG
**D-GLP-1_diff_Acetylated**	Acetyl-**HE**AS**TF**A**D**SAA**Y**TNAKAAA**IF**WA**L**ARLL
**D-GLP-1_mirror_image_search**	RGKAF**L**EL**FI**LAAGELIEAI**D**R**TF**TG**E**A**H**

[a] The hotspots and matched residues in L-GLP-1 and the D-peptides are highlighted in bold. The hotspot residues selected to design both peptides are highlighted in yellow. Y19, which was only chosen as a hotspot to design D-GLP-1_diff_Acetylated, is highlighted in green.

A few minor suggestions:

1) in FigS2, the colors for % range don't seem to match; it would be easier to compare if they are the same.

It was corrected.

2) There is a typo on Page 7. The last mention of helix2 should be helix 3.

It was corrected.

3) Figure 3 has no legend for H. Also it is not clear where licorice color is shown, just labels?

It was corrected

Additional Questions:

Quality of experimental data, technical rigor: Moderate

Significance to chemistry researchers in this and related fields: High

Broad interest to other researchers: High

Novelty: Moderate

Is this research study suitable for media coverage or a First Reactions (a News & Views piece in the journal)?: No

oc-2023-01488a.R2

Name: Peer Review Information for "HelixDiff a Score-based Diffusion Model for Generating All-atom Alpha Helical Structures"

Second Round of Reviewer Comments

Reviewer: 1

Comments to the Author

The authors have satisfyingly addressed the concerns I had raised.

Author's Response to Peer Review Comments:

Philip M. Kim, Ph.D.
Professor, The Donnelly Centre for Cellular + Biomolecular Research
Department of Molecular Genetics
Department of Computer Science
University of Toronto | www.kimlab.org | thedonnellycentre.utoronto.ca

Editor
ACS Central Science

Toronto, March 19th, 2024

Dear Editor:

Please find attached a revised version of our manuscript, "*HelixDiff a Score-based Diffusion Model for Generating All-atom Alpha Helical Structures*." We thank you for your careful consideration and provisional acceptance of our manuscript. We have addressed all the format requirements highlighted in the decision letter; no other changes were included in this revised version.

With best regards,

Philip M. Kim, Ph.D.
